# The Impact of Host Abundance on the Epidemiology of Tick-Borne Infection

**DOI:** 10.1007/s11538-023-01133-8

**Published:** 2023-03-09

**Authors:** Xander O’Neill, Andy White, Christian Gortázar, Francisco Ruiz-Fons

**Affiliations:** 1 Maxwell Institute for Mathematical Sciences and Department of Mathematics, Heriot-Watt University, Edinburgh, EH14 4AS UK; 2 SaBio, Instituto de Investigación en Recursos Cinegéticos IREC (UCLM & CSIC), 13005 Ciudad Real, Spain

**Keywords:** Mathematical modelling, Tick-host models, Tick-borne pathogens, Zoonotic spillover

## Abstract

Tick-borne diseases are an increasing global public health concern due to an expanding geographical range and increase in abundance of tick-borne infectious agents. A potential explanation for the rising impact of tick-borne diseases is an increase in tick abundance which may be linked to an increase in density of the hosts on which they feed. In this study, we develop a model framework to understand the link between host density, tick demography and tick-borne pathogen epidemiology. Our model links the development of specific tick stages to the specific hosts on which they feed. We show that host community composition and host density have an impact on tick population dynamics and that this has a consequent impact on host and tick epidemiological dynamics. A key result is that our model framework can exhibit variation in host infection prevalence for a fixed density of one host type due to changes in density of other host types that support different tick life stages. Our findings suggest that host community composition may play a crucial role in explaining the variation in prevalence of tick-borne infections in hosts observed in the field.

**Supplementary Information:**

The online version contains supplementary material available at 10.1007/s11538-023-01133-8.

## Introduction

Ticks are a parasitic invertebrate, of the class *Arachnida*, that survive and progress through their life-stages, i.e. larvae, nymph, and adult, by feeding on the blood of vertebrate animals (Cupp 1991). Progression between tick life-stages requires at least one blood meal. Larva and nymphs of exophilic/non-nidicolous ixodid ticks generally feed on smaller hosts, such as rabbits (*Oryctolagus cuniculus*), hares (*Lepus* spp.), reptiles, or birds; and adults generally feed on larger hosts, such as red deer (*Cervus elaphus*), wild boar (*Sus scrofa*) or livestock. Nymphs can often feed on either small or large hosts, although the exact connection between tick life-stage and the hosts on which they feed can vary between tick species (Anderson and Magnarelli 2008). The interaction between ticks and hosts can lead to the exchange of infectious pathogens potentially causing disease in hosts (Rahlenbeck et al. 2016). Ticks can become infected with pathogens through feeding on an infected host and can then transmit the infection by feeding on susceptible hosts at a later point in their life cycle (Shi et al. 2018). Ticks can also become infected through vertical transmission from infected adults to eggs, by co-feeding (where a susceptible tick feeds with an infected tick on the same host) or even during adult mating (Ferreri et al. 2016; Moore et al. 2018).

At the global scale ticks transmit the largest range of infectious diseases of all arthropod vectors (Jongejan and Uilenberg 2004). They can cause severe toxic conditions such as paralysis and toxicosis, and along with their pathogens, have co-evolved with wildlife hosts which act as reservoirs for the ticks and tick-borne infections (Jongejan and Uilenberg 2004). Tick-borne infections are an increasing global public health concern due to the increasing abundance of ticks, expanding geographical range for vectors and pathogens, and emerging tick-borne infectious agents (Gortázar et al. 2014; Jones et al. 2008; Mysterud et al. 2018). There is evidence that this increased threat is linked to climate change that has made environmental conditions more favourable to ticks (Bouchard et al. 2019; Gortázar et al. 2014; Randolph 2008). It has also been suggested that increases in the abundance and range of wildlife reservoir hosts may play an important role in the increased risk of spillover of tick-borne disease to humans and livestock (Gortázar et al. 2014; Michelitsch et al. 2019; Mysterud et al. 2018).

A potential explanation for the increasing impact of tick-borne infection is an increase in tick abundance (Mysterud et al. 2018; Scharlemann et al. 2008). Host community composition can limit and regulate tick abundance which can have a profound impact on the incidence of tick-borne infection (Cobbold et al. 2015). However, there is considerable variation in the incidence of infection at a regional level, which may be affected by the abundance and competence of specific vertebrate hosts. We also know little about the link between host community composition and tick abundance (Cobbold et al. 2015; Mysterud et al. 2018). Some studies indicate a direct link between host diversity and infection risk (Begon 2008; Johnson and Thieltges 2010) and some indicate that increased host biodiversity could lead to increased parasite diversity and exposure (Jones et al. 2008). Field evidence suggests that there has been a widespread increase in the density of wild ungulates across Europe (Apollonio et al. 2010; Massei et al. 2015; Milner et al. 2006). Since these are key hosts for ticks this could have an impact on tick demography with a consequent impact on the prevalence and risk of zoonotic transmission of tick-borne infectious disease. However, ticks may require different hosts to complete their development and so without a commensurate increase in other hosts, such as small mammals, there may be a bottleneck in tick development that limits tick density increases (Cobbold et al. 2015). This could have important consequences for tick-borne infections. The aim of our study is to develop mathematical models to assess the impact of host density on tick demography and the epidemiological dynamics of tick-borne pathogens.

Mathematical models have been used to explore and understand the epidemiological dynamics of host-tick interactions and the persistence of tick-borne infections (Lou and Wu 2014; Norman et al. 1999; Rosá et al. 2003; Rosá and Pugliese 2007; Switkes et al. 2016). Switkes et al. (2016) (Switkes et al. 2016) developed a model for Crimean-Congo haemorrhagic fever virus (CCHFv) that considered a single tick class (all tick stages were grouped into one class) and two large mammal hosts (cattle and humans). This model considered a constant tick population size, with no link between host density and tick density. They suggested tick-human transmission as the main cause for the spread of CCHFv in humans. Norman et al. 1999 (Norman et al. 1999) developed a model (later extended by Rosá et al. (2003) (Rosá et al. 2003)) to understand the persistence of the Louping-ill virus in grouse. They presented a stage-structured model for ticks where all stages could feed on either a viraemic or non-viraemic host and tick birth rate was linked to host density but was also limited through self-regulation. Results showed that non-viraemic hosts could either amplify the tick population and assist in virus persistence or dilute the infection and cause it to die out. Although these studies considered the link between ticks and multiple hosts on tick density, they have not considered the important link between different tick stages and the different hosts on which they feed. Notwithstanding, these studies do highlight that the link between host and tick density can be critical to explaining the epidemiology of tick-borne infections.

Model frameworks have been developed where different tick stages can feed on different hosts (Lou and Wu 2014; Rosá and Pugliese 2007). Lou and Wu (2014) (Lou and Wu 2014) developed a model where larvae and nymphs could feed on small mammals, adults on large mammals, and where progression between stages depended on an attachment term that was a function of the respective hosts. The infection dynamics considered direct transmission between ticks and small mammals only. It was shown that tick infection risk could decrease at high small mammal density due to a decrease in total births but an increase in larvae/nymph progression rate. Rosá and Pugliese (2007)(Rosá and Pugliese 2007) developed a general model to explore the effect of tick population dynamics and host density on the persistence of tick-borne infections. The dynamic regulation of tick density was included through a density-dependent birth or density-dependent moulting term, with density-dependent moulting representing how increases in host immunity could limit moulting success (Brossard and Wikel 2004; Wikel 1996). They showed that the effect of host densities on tick-borne infection dynamics depended strongly on how the tick population is regulated and that the dilution effect of an infection for a high density of competent hosts occurred only if tick density is independent of host density. In these model frameworks tick reproduction and development depended on host health that was averaged across all hosts. They did not consider how the health of specific hosts would affect the specific tick stages that feed on them. Furthermore, the density-dependent functions used a negative exponential form which unrealistically led to a decrease in the production of larvae as tick density increased. Pugliese and Rosá (2008) (Pugliese and Rosá 2008) replaced the negative exponential density-dependent functions with a Holling type-II form but here regulation through host density was no longer examined. Therefore, studies that have used mathematical models to examine the epidemiological dynamics of tick-borne infections have either not considered the link between different tick stages and the different hosts on which they feed or have not considered how tick attachment would be dependent on tick and host density.

In this study we will develop a model framework that links tick attachment, and therefore progression to the next tick life-stage, to the specific hosts on which the different tick stages feed. Our model framework will include separate questing and feeding classes of ticks. Since the density of infected questing ticks is linked to spillover (Klaus et al. 2010) this will allow us to examine whether host community composition is related to the risk of zoonotic infection in humans and livestock. Our model will also include regulation of tick density due to host availability, where at high questing tick density and fixed host density there is a limit on the number of ticks that can attach onto a host. Importantly, this regulation will be dependent on the density of the specific questing tick stage and the density of the specific hosts on which they feed (Mysterud et al. 2018; Cobbold et al. 2015). Our aim is to understand the impact of host community composition (variation of both host density and host composition) on the density of ticks and the persistence, prevalence, and risk of transmission of tick-borne infections. We consider two host types within our modelling approach; smaller hosts, that can support larval and nymphal tick stages, and larger hosts, that can support nymphal and adult tick stages. We also consider changes in host composition through the variation of the density of the different host types and through the variation of the parasitisation index of the different host types (the average number of ticks a host can harbour). The latter is important as different host species can support different tick burdens and host species composition may differ at the regional scale (LoGuidice et al. 2003). This paper is separated into a demographic modelling section, which examines the effect of host community composition on the densities of ticks in the absence of infection, and an epidemiological modelling section, where the demographic model is expanded to explore the effect of host community composition on the infection dynamics in ticks and hosts.

## Tick-Host Demographic Dynamics

We outline a model that represents the tick-host demographic dynamics. Following the framework of (Rosá and Pugliese 2007), we consider a stage-structured model for larval, *L*, nymph, *N*, and adult, *A*, ticks that also differentiates between questing (subscript *Q*) and feeding (subscript *F*) ticks. For our model framework the questing class includes time spent egg laying, hatching, moulting and diapause as appropriate for each life stage. We assume specific tick stages can feed on specific hosts, with larvae and nymphs able to feed on small hosts, $H_S$ and nymphs and adults able to feed on large hosts, $H_L$. Note, we partition the feeding nymph stage depending on whether they feed on small hosts, $N_{FS}$ or large hosts $N_{FL}$. The model for the tick demographic dynamics is described by the following equations:

1 $$\begin{aligned}&\frac{\textrm{d}L_Q}{dt} = \sigma _Aa_TA_F - \beta _1\Phi _1(L_Q, N_Q)H_SL_Q - b_LL_Q,\nonumber \\&\frac{\textrm{d}L_F}{dt} = \beta _1\Phi _1(L_Q, N_Q)H_SL_Q - \sigma _LL_F,\nonumber \\&\frac{\textrm{d}N_Q}{dt} = \sigma _LL_F - \beta _2\Phi _1(L_Q, N_Q)H_SN_Q - \beta _3\Phi _2(N_Q, A_Q)H_LN_Q - b_NN_Q,\nonumber \\&\frac{\textrm{d}N_{FS}}{\textrm{d}t} = \beta _2\Phi _1(L_Q, N_Q)H_SN_Q - \sigma _NN_{FS},\nonumber \\&\frac{\textrm{d}N_{FL}}{\textrm{d}t} = \beta _3\Phi _2(N_Q, A_Q)H_LN_Q - \sigma _NN_{FL}, \nonumber \\&\frac{\textrm{d}A_Q}{\textrm{d}t} = \sigma _NN_{FS} +\sigma _NN_{FL} - \beta _4\Phi _2(N_Q, A_Q)H_LA_Q - b_AA_Q, \nonumber \\&\frac{\textrm{d}A_F}{\textrm{d}t} = \beta _4\Phi _2(N_Q, A_Q)H_LA_Q - \sigma _AA_F.\end{aligned}$$

2 $$\begin{aligned}&\qquad \Phi _1(L_Q, N_Q) = \frac{1}{1 + s_1L_Q+s_2N_Q}, \quad \Phi _2(N_Q, A_Q) = \frac{1}{1 + s_3N_Q+s_4A_Q}. \end{aligned}$$

Here, $a_T$ represents the average number of larvae produced per adult tick, and $b_L, b_N$ and $b_A$ represent the natural death rates of larvae, nymphs, and adults, respectively. We assume that attachment depends on the specific host on which the specific ticks feed. The attachment coefficients are given by $\beta _1, \beta _2, \beta _3$ and $\beta _4$, where attachment depends on the relative density of the number of questing ticks that can attach to the specific host and is controlled by the coefficients $s_1$ to $s_4$ (see Eqs. 2). The attachment process can be considered as competition between the relevant questing tick classes, where increases in questing tick density leads to an increase in attachment and where the attachment rates saturate due to limitations in the number of ticks that a host can harbour. Previous model studies have shown that the relationship between ticks and hosts can vary and is not necessarily linear with host density (Ostfeld 2010). Lou and Wu (2017) approximated this variation by using density-dependent attachment at low host density, and frequency-dependent attachment at high host density (Lou and Wu 2017). Whilst our model formulation does not explicitly include a frequency-dependent/density-dependent relationship, it does implicitly capture this dynamic. In our model, having a fixed density of one host type whilst varying the other results in density-dependent style attachment at low density of the varying host. At high density of the varying host type, the density of the fixed host causes a bottleneck in tick progression and so tick density begins to saturate, resulting in frequency-dependent style attachment. Ticks detach from hosts at rates $\sigma _L$, $\sigma _N$ and $\sigma _A$ (Valcárcel et al. 2016) (and the reciprocal of these rates represent the average feeding times of the different tick stages). The model and parameters values (excluding the attachment coefficients) are representative of a general three-host tick, such as *Hyalomma lusitanicum* and *Ixodes ricinus*, as detailed in Valcárcel et al. (2016) and Matser et al. (2018) (Valcárcel et al. 2016; Matser et al. 2009) and are outlined in Table .

**Table 1:** Definitions and baseline values for the demographic parameters. For references see main text

Parameter	Description (rates given per day)	Value
$a_T$	Average number of larvae produced per adult tick	1500
$b_L$	Natural death rate of questing larvae	0.017
$b_N$	Natural death rate of questing nymph	0.00519
$b_A$	Natural death rate of questing adults	0.0027
$\sigma _L$	Rate of progression from $L_f$ to $N_q$	1/6
$\sigma _N$	Rate of progression from $N_f$ to $A_q$	1/11
$\sigma _A$	Rate of progression from $A_f$ to $L_q$	1/17
$\beta _1$	Attachment coefficient for larvae onto small hosts	0.00000049
$\beta _2$	Attachment coefficient for nymph onto small hosts	0.000033
$\beta _3$	Attachment coefficient for nymph onto large hosts	0.000014
$\beta _4$	Attachment coefficient for adults onto large hosts	0.00035
$s_{1}$	Coefficient for the regulation of attachment of larvae to small hosts	0.00000015
$s_{2}$	Coefficient for the regulation of attachment of nymphs to small hosts	0.000018
$s_{3}$	Coefficient for the regulation of attachment of nymphs to large hosts	0.000002
$s_{4}$	Coefficient for the regulation of attachment of adults to large hosts	0.000074

The parametrisation of the attachment coefficients $\beta _1, \beta _2, \beta _3$ and $\beta _4$ and the natural death rates $b_L, b_N$ and $b_A$ is determined by using data on the average duration a *Hyalomma lusitanicum* tick would spend in each class (Valcárcel et al. 2016). Under laboratory conditions, a *Hyalomma lusitanicum* tick would spend approximately 15 days as a questing larvae, 6 days as a feeding larvae, 29 days as a questing nymph (including the moulting phase from larvae to nymph), 11 days as a feeding nymph, 42 days as a questing adult (including the moulting phase from nymph to adult) and 17 days as a feeding adult (Valcárcel et al. 2016). However, under field conditions the tick lifespan is expected to take approximately one year to complete (Valcárcel et al. 2016). Assuming that the duration spent feeding is constant, we scale the durations spent as a questing tick under laboratory conditions to approximate the length of time spent in each questing class in the environment. As such, we take the average duration spent in the questing larvae class (which is given by the expression $1/(\beta _1\Phi _1 H_s + b_L)$) to be 58 days, questing nymph class ($1/(\beta _2\Phi _1 H_s + \beta _3\Phi _2 H_u + b_N)$) to be 112 days and questing adult class ($1/(\beta _4\Phi _2 H_u + b_A)$) to be 162 days, which gives an overall, average life cycle duration of one year (Valcárcel et al. 2016). We fit these average durations at baseline host densities of $H_S = 200/km^2$ for small hosts and $H_L = 20/km^2$ for large hosts (Carro et al. 2019; LoGuidice et al. 2003; Acevedo et al. 2007, 2008). For the parasitisation index levels of ticks on hosts we consider an average of 10 ticks per small host and 40 ticks per large host (Dwużnik-Szarek et al. 2021; LoGuidice et al. 2003; Vor et al. 2010; Ruiz-Fons et al. 2013). The attachment coefficient values are given in Table 1. Note, whilst the parametrisation of these coefficients and rates are based on a *Hyalomma lusitanicum* tick, with an average life cycle duration of one year, the model can be fitted to ticks with different life cycle durations. The model results are qualitatively similar and our key findings remain unchanged (results not shown).

### The Impact of Host Density on Tick Density

We vary the density of small and large hosts to see the impact on questing and feeding tick densities (Fig. ). For fixed large host densities, increases in small host density leads to increases in the density of all tick classes, except for nymphs feeding on large hosts, which see an initial increase and then a decrease in density (Fig. ). The density of adult ticks feeding on large hosts increases and saturates at high small host density due to the limited availability of large hosts. Consequently, the production of questing larvae increases and then saturates. For nymphs feeding on large hosts initial increases in small host density result in an increase in questing nymph density and so increases the density of nymphs feeding on large hosts. However, at high small host densities there is an increased relative availability of small hosts compared to large hosts, which results in a decrease in the density of nymphs feeding on large hosts (Fig. 2B(ii)). Note, at average baseline host densities, the density of questing larvae is greater than the density of questing nymphs, which is greater than the density of questing adults. However, for high small host density, progression through the larvae and nymph stage is more rapid due to a high relative number of small hosts for attachment. Attachment for adult ticks is limited, due to the low relative density of large hosts, and this leads to an increase in density of questing adult ticks. A result is that the steady state density of adult ticks may exceed the steady state density of questing nymphs (but this should not be interpreted as nymphs producing more than one adult tick).

**Fig. 1:**
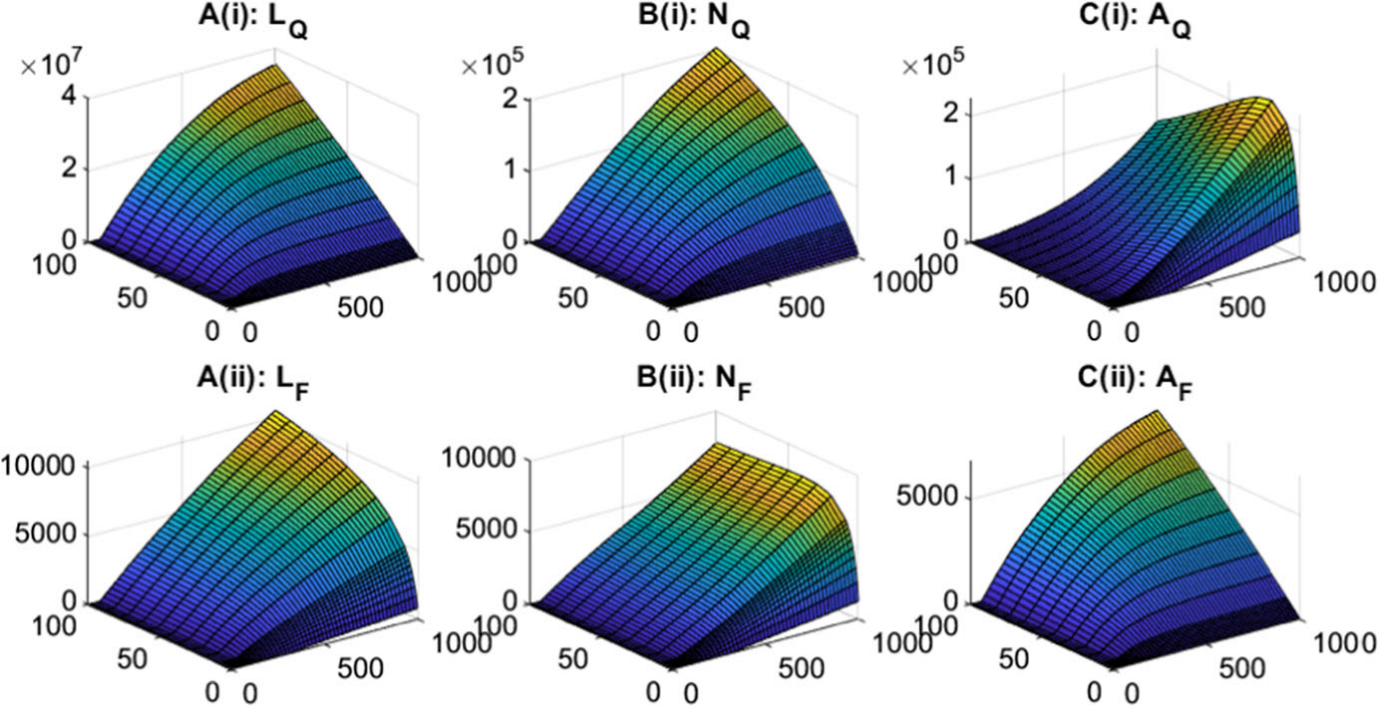
Tick population densities (vertical axis) for a varying large host density (from 0 to 100) and varying small host density (from 0 to 1000) for the tick-host model framework represented by Eqs. 1–2. Steady state densities were plotted for **A** larvae, **B** nymphs and **C** adults, for both (i) questing ticks and (ii) feeding ticks. When not varied in the figure parameter values are as in Table 1. Note, the axes scales may differ between subplots

**Fig. 2:**
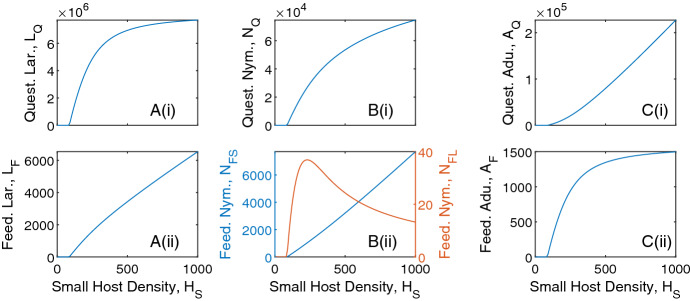
Tick population densities for a varying small host density and fixed large host density, $H_u = 20$, for the tick-host model framework represented by Eqs. 1–2. Steady state densities were plotted for **A** larvae, **B** nymphs and **C** adults, for both (i) questing ticks and (ii) feeding ticks. When not varied in the figure parameter values are as in Table 1. Note, the axes scales may differ between subplots

**Fig. 3:**
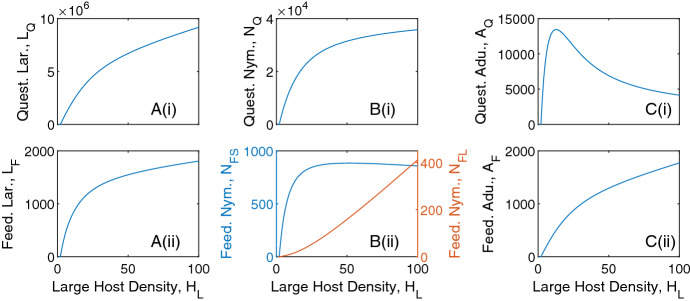
Tick population densities for a varying large host density and fixed small host density, $H_S = 200$, for the tick-host model framework represented by Eqs. 1–2. Steady state densities were plotted for **A** larvae, **B** nymphs and **C** adults, for both (i) questing ticks and (ii) feeding ticks. When not varied in the figure parameter values are as in Table 1. Note, the axes scales may differ between subplots

Similarly, for fixed small host densities, an increase in large host density increases the density of all tick classes, except for nymphs feeding on small hosts and questing adult ticks which see an initial increase then a decrease in density (Fig. ). The density of larvae feeding on small hosts (and subsequently, the density of questing nymphs) increases and saturates as the large host density increases, due to the limited availability of small hosts. Initial increases in large host density increase the density of feeding nymphs on small hosts due to increased questing nymph abundance. However, at high large host density there is an increased relative availability of large hosts compared to small hosts, which results in a decrease in the density of nymphs feeding on small hosts. There is an initial increase in questing adult tick density as large host density increases due to the increase in total feeding nymph density, but further increases in large host density leads to increased availability of hosts for feeding adult ticks and so decreases the density of questing adult ticks.

A key feature of our model framework is that the tick population density is regulated due to the limitation on tick attachment to specific hosts (Cobbold et al. 2015). In particular, host density is a determinant of the density of the different questing and feeding tick classes and this will have important consequences for the epidemiological dynamics. This behaviour, in which feeding tick density saturates for fixed density of one host as the density of the other host increases, was not exhibited in previous studies that developed tick demographic models to examine the persistence and dynamics of tick-borne infections (Lou and Wu 2014; Rosá and Pugliese 2007).

### The Impact of Host Density on Tick Density Under Different Parasitisation Index Levels

Regional differences in host species composition can lead to variation in the average parasitisation index of hosts. In North America, for example, raccoons and skunks can act as large hosts and will have a difference average tick burden to ungulate hosts in Spain, such as wild boar and red deer (LoGuidice et al. 2003; Ruiz-Fons et al. 2013). We consider parasitisation index levels of 5, 10 and 20 per small host ($PI_S$) and 5, 40 and 80 per large host ($PI_L$), and recalibrate the values of the attachment coefficients $\beta _1, \beta _2, \beta _3, \beta _4$ and $s_1, s_2, s_3, s_4$, for each $PI_S$ and $PI_L$. We vary small host density at fixed large host density for five different sets of parasitisation index levels (Figures S.1 - S.5) and vary large host density at fixed small host density for the same five sets of parasitisation index levels (Figures S.6 - S.10).

In general, we find that the qualitative behaviour for increases in small and large hosts (Figs. 1–3) holds for different parasitisation index levels. However, some quantitative differences are apparent. As either parasitisation index increases the overall tick density increases. For a higher ratio of $PI_S:PI_L$ there is a more rapid saturation of tick densities when the density of small hosts is increased, since the limitation due to available large hosts is realised at lower small host densities. Similarly, for a higher ratio of $PI_L:PI_S$ there is a more rapid saturation in tick densities when the density of large hosts is increased. For further details see section S.1.

## Tick-Host Epidemiological Dynamics

We extend our model of host-tick dynamics where each tick stage feeds on specific hosts (Eqs. 1-2) to include a representation of tick-borne infections. Previous studies that have examined mathematical models of tick-borne infections have determined thresholds for pathogen persistence (Hudson et al. 1995) and explored how different host types (Norman et al. 1999; Rosá and Pugliese 2007; Rosá et al. 2003; Switkes et al. 2016) and seasonality (Lou et al. 2014; Roome et al. 2018) will affect the epidemiological dynamics. Models have shown that increases in host density or host diversity can either have a diluting effect or an amplifying effect on the persistence of tick-borne infections (Levi et al. 2016; Norman et al. 1999; Rosá et al. 2003). Our study will examine the epidemiological dynamics for a host-tick model in which tick density is dependent on host community composition (in terms of host density and host parasitisation index levels) and where tick density can saturate due to limited availability of one host type. This will allow us to assess how changes in host community composition can impact the prevalence and risk of spillover of tick-borne infectious disease. Our model will include the key transmission routes for tick-borne infections. These include tick to host and host to tick direct transmission, a non-systemic (co-feeding) transmission route and vertical transmission from parent to offspring (Ferreri et al. 2016; Nonaka et al. 2010). Although important in some circumstances (Norman et al. 1999; Rosá et al. 2003), non-infectious hosts are omitted and we assume all hosts can become infected and infectious. The general model framework we present can be adapted to explore the epidemiological dynamics of many different tick-borne disease systems.

To extend the demographic model (Eqs. 1-2) each tick stage and host type are split into a susceptible class (additional subscript *s*) or an infected class (additional subscript *i*) and for the hosts, a recovered and immune class (additional subscript *r*). The full model is detailed for the tick dynamics (Eqs. 3), small host dynamics (Eqs. 4) and large host dynamics (Eqs. 5).

3 $$\begin{aligned}&\frac{dL_{Qs}}{\textrm{d}t} = a_T\sigma _A\left( A_{Fs} + (1-\rho )A_{Fi}\right) - \beta _1\Phi _1H_SL_{Qs} - b_LL_{Qs},\nonumber \\&\frac{dL_{Qi}}{\textrm{d}t} = a_T\sigma _A\rho A_{Fi} - \beta _1\Phi _1H_SL_{Qi} - b_LL_{Qi}, \nonumber \\&\frac{dL_{Fs}}{\textrm{d}t} = \beta _1\Phi _1\left( H_S - p^LH_{Si}\right) L_{Qs} - \theta _{TT}\frac{I_{TS}}{T_{TS}}L_{Fs} - \sigma _LL_{Fs}, \nonumber \\&\frac{dL_{Fi}}{\textrm{d}t} = \beta _1\Phi _1H_SL_{Qi} + p^L\beta _1\Phi _1H_{Si}L_{Qs} +\theta _{TT}\frac{I_{TS}}{T_{TS}}L_{Fs} - \sigma _LL_{Fi}, \nonumber \\&\frac{\textrm{d}N_{Qs}}{\textrm{d}t} = \sigma _LL_{Fs} - \beta _2\Phi _1H_SN_{Qs} - \beta _3\Phi _2H_LN_{Qs} - b_NN_{Qs}, \nonumber \\&\frac{\textrm{d}N_{Qi}}{\textrm{d}t} = \sigma _LL_{Fi} - \beta _2\Phi _1H_SN_{Qi} - \beta _3\Phi _2H_LN_{Qi} - b_NN_{Qi}, \nonumber \\&\frac{\textrm{d}N_{FSs}}{\textrm{d}t} = \beta _2\Phi _1\left( H_S - p^{N_1}H_{Si}\right) N_{Qs} -\theta _{TT}\frac{I_{TS}}{T_{TS}}N_{FSs} -\sigma _NN_{FSs},\nonumber \\&\frac{\textrm{d}N_{FSi}}{\textrm{d}t} = \beta _2\Phi _1H_SN_{Qi} + p^{N_1}\beta _2\Phi _1H_{Si}N_{Qs} +\theta _{TT}\frac{I_{TS}}{T_{TS}}N_{FSs} - \sigma _NN_{FSi},\nonumber \\&\frac{\textrm{d}N_{FLs}}{\textrm{d}t} = \beta _3\Phi _2\left( H_L - p^{N_2}H_{Li}\right) N_{Qs} -\theta _{TT}\frac{I_{TL}}{T_{TL}}N_{FLs} - \sigma _NN_{FLs},\nonumber \\&\frac{\textrm{d}N_{FLi}}{\textrm{d}t} = \beta _3\Phi _2H_LN_{Qi} + p^{N_2}\beta _3\Phi _2H_{Li}N_{Qs} +\theta _{TT}\frac{I_{TL}}{T_{TL}}N_{FLs} - \sigma _NN_{FLi},\nonumber \\&\frac{\textrm{d}A_{Qs}}{\textrm{d}t} = \sigma _NN_{FSs} + \sigma _NN_{FLs} -\beta _4\Phi _2H_LA_{QS} -b_AA_{Qs}, \nonumber \\&\frac{\textrm{d}A_{Qi}}{\textrm{d}t} = \sigma _NN_{FSi} + \sigma _NN_{FLi} -\beta _4\Phi _2H_LA_{Qi} -b_AA_{Qi}, \nonumber \\&\frac{\textrm{d}A_{Fs}}{\textrm{d}t} = \beta _4\Phi _2\left( H_L - p^AH_{Li}\right) A_{Qs} -\theta _{TT}\frac{I_{TL}}{T_{TL}}A_{Fs} - \sigma _AA_{Fs},\nonumber \\&\frac{\textrm{d}A_{Fi}}{\textrm{d}t} = \beta _4\Phi _2H_LA_{Qi} + p^A\beta _4\Phi _2H_{Li}A_{Qs} +\theta _{TT}\frac{I_{TL}}{T_{TL}}A_{Fs} - \sigma _AA_{Fi}.\end{aligned}$$

4 $$\begin{aligned}&\frac{\textrm{d}H_{Ss}}{\textrm{d}t} = a_SH_S\left( 1 - q_SH_S\right) - \left( q^L\beta _1\Phi _1L_{Qi} + q^{N1}\beta _2\Phi _1N_{Qi}\right) H_{Ss} - d_SH_{Ss},\nonumber \\&\frac{\textrm{d}H_{Si}}{\textrm{d}t} = \left( q^L\beta _1\Phi _1L_{Qi} + q^{N1}\beta _2\Phi _1N_{Qi}\right) H_{Ss} - \gamma _SH_{Si} - d_SH_{Si},\nonumber \\&\frac{\textrm{d}H_{Sr}}{\textrm{d}t} = \gamma _SH_{Si} - d_SH_{Sr}.\end{aligned}$$

5 $$\begin{aligned}&\frac{dH_{Ls}}{\textrm{d}t} = a_LH_L\left( 1 - q_LH_L\right) - \left( q^{N2}\beta _3\Phi _2N_{Qi} + q^{A}\beta _4\Phi _2A_{Qi}\right) H_{Ls} - d_LH_{Ls},\nonumber \\&\frac{dH_{Li}}{\textrm{d}t} = \left( q^{N2}\beta _3\Phi _2N_{Qi} + q^{A}\beta _4\Phi _2A_{Qi}\right) H_{Ls} - \gamma _LH_{Li} - d_LH_{Li},\nonumber \\&\frac{\textrm{d}H_{Lr}}{\textrm{d}t} = \gamma _LH_{Li} - d_LH_{Lr}. \end{aligned}$$

Here, $H_S = H_{Ss} + H_{Si} + H_{Sr}$ and $H_L = H_{Ls} + H_{Li} + H_{Lr}$ denote the total population density of small and large hosts, respectively, $I_{TS} = L_{Fi} + N_{FSi}$ and $I_{TL} = N_{FLi} + A_{Fi}$ represent the total infected ticks feeding on small and large hosts, respectively, and $T_{TS} = L_{Fs} + L_{Fi} + N_{FSs} + N_{FSi}$ and $T_{TL} = N_{FLs} + N_{FLi} + A_{Fs} + A_{Fi}$ represent the total ticks feeding on small and large hosts, respectively. We assume hosts have natural death rates $d_S, d_L$ and maximum birth rates $a_S, a_L$ which are modified through self-regulation using the parameters $q_S, q_L$, leading to a carrying capacity of $K_S = (a_S-d_S)/(a_Sq_S)$ and $K_L = (a_L - d_L)/(a_Lq_L)$, for small and large hosts, respectively. The terms representing regulation through attachment, $\Phi _1$ and $\Phi _2$ are the same as in Eq. (2) with $L_Q = L_{Qs} + L_{Qi}, N_Q = N_{Qs} + N_{Qi}$ and $A_Q = A_{Qs} + A_{Qi}$ denoting the total questing larvae, nymph and adult population densities, respectively.

**Table 2:** Definitions and baseline values for the epidemiological parameters and host demographic parameters

Parameter	Description (rates given per day)	Value
$p^L$	Infected host to larvae transmission coefficient	0.345
$p^{N1}, p^{N2}$	Infected host to nymph transmission coefficient	0.36
$p^A$	Infected host to adult transmission coefficient	0.22
$q^L, q^{N1}$	Infected tick to small host transmission coefficient	0.25
$q^{N2}, q^A$	Infected tick to large host transmission coefficient	0.0125
$\rho$	Vertical transmission proportion	0.15
$\theta _{TT}$	Non-systemic transmission coefficient (co-feeding)	0.025
$\gamma _S$	Recovery rate of infected small hosts	1/14
$\gamma _L$	Recovery rate of infected large hosts	1/7
$PI_S$	Parasitisation index for small hosts	10
$PI_L$	Parasitisation index for large hosts	40
$a_S$	Maximum birth rate of small hosts	0.05
$a_L$	Maximum birth rate of large hosts	$\log (4)/365$
$d_S$	Natural death rate of small hosts	1/365
$d_L$	Natural death rate of large hosts	1/1460
$K_S$	Carrying capacity for small hosts	200
$K_L$	Carrying capacity for large hosts	20

We assume four methods of transmission: host to tick, tick to host, vertical transmission, and non-systemic transmission (co-feeding) (see Figure S.11 in the supplementary material). The first three transmission routes are as described in Rosá and Pugliese (2007) (Rosá and Pugliese 2007) with host-tick transmission coefficients $p^L, p^{N1}, p^{N2}$ and $p^A$ for each respective class of tick; tick to host transmission coefficients $q^L, q^{N1}, q^{N2}$ and $q^A$ for each respective class of tick and for vertical transmission a proportion $\rho$ of larvae reproduced from feeding infected adult ticks are infected. Ticks acquire infection through non-systemic transmission (co-feeding ticks) during feeding with transmission coefficient $\theta _{TT}$ and at a rate dependent on the ratio between infected ticks and total ticks feeding on a specific host. Co-feeding transmission can occur between a susceptible and infected tick of any tick class that feeds on the same type of host. Small and large hosts can recover from the infection at rates $\gamma _S$ and $\gamma _L$, respectively.

We choose parameter values (see Table ) which, where possible, resemble tick-borne infection transmission data (Matser et al. 2009) and host population dynamics (Barasona et al. 2016; González-Barrio et al. 2015; Villafuerte 2019). We choose tick-host transmission coefficients, representative of a general form of tick-borne infection, such that infection persists in all hosts and ticks and such that the seroprevalence in both host types was approximately $30-40\%$ for baseline parameter values, which has been seen in field situations (Fajs et al. 2014; Lihou et al. 2020). These parameter values are not intended to represent a specific tick-borne infection but will allow the trends in the epidemiological dynamics to be assessed as host community composition changes. We also undertake a sensitivity analysis to explore the impact of different epidemiological parameter choices on the epidemiological dynamics (see Sect. 3.3).

### The Impact of Host Density on Tick and Host Pathogen Epidemiology

**Fig. 4:**
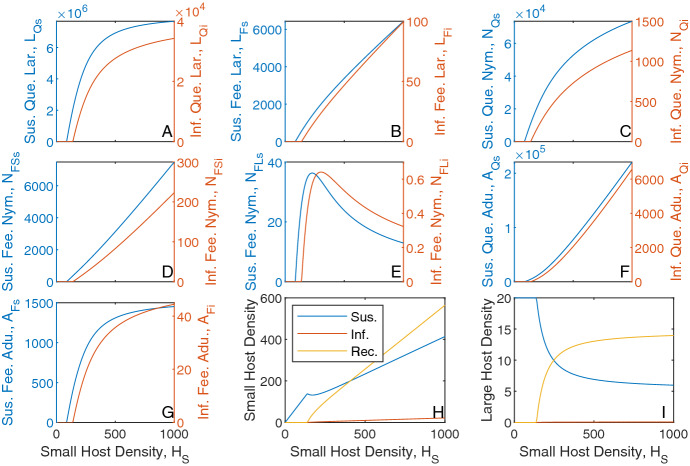
Population densities for a varying small host density and fixed large host density, $H_L = 20$, for the tick-host model framework with infection represented by Eqs. 3–5. Steady state densities were plotted for **A** questing larvae, **B** feeding larvae, **C** questing nymph, **D** feeding nymphs on small hosts, **E** feeding nymphs on large hosts, **F** questing adults, **G** feeding adults, **H** small hosts and **I** large hosts. Each plot indicates the infection status of individuals, with (*blue*) susceptible, (*orange*) infected and (*yellow*) recovered. When not varied in the figure parameter values are as in *Table*1 and 2. Note, the axes scales may differ between subplots (Color figure online)

For the model with infection, when the small or large host density is increased the total tick and susceptible tick densities exhibit similar trends to those seen for the model in the absence of infection (compare Figs. 2–3 with Figs. –). For a fixed large host density, low levels of small host density cannot sustain the infection (Fig. 4). With increases in small host density the infection persists and the density of each infected tick stage follow similar trends to those seen for the respective susceptible classes. Increases in small host density results in an increased density of infected questing ticks, which increases the contact rate between infected ticks and small hosts and consequently increases the density of infected and recovered small hosts. As the large host density remains fixed there is a limit in the total number of ticks that can attach to large hosts, and so the infected and recovered large host densities increase and saturate as small host densities increase to high levels.

**Fig. 5:**
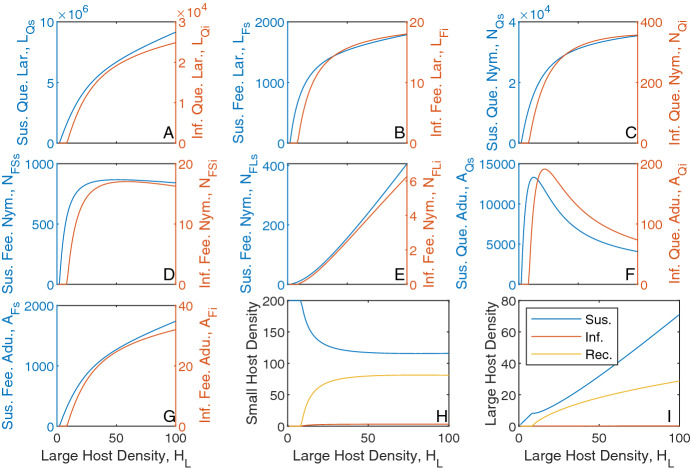
Population densities for a varying large host density and fixed small host density, $H_S = 200$, for the tick-host model framework with infection represented by Eqs. 3–5. Steady state densities were plotted for **A** questing larvae, **B** feeding larvae, **C** questing nymph, **D** feeding nymphs on small hosts, **E** feeding nymphs on large hosts, **F** questing adults, **G** feeding adults, **H** small hosts and **I** large hosts. Each plot indicates the infection status of individuals, with (*blue*) susceptible, (*orange*) infected and (*yellow*) recovered. When not varied in the figure parameter values are as in Table 1 and 2. Note, the axes scales may differ between subplots (Color figure online)

Similarly, for a fixed small host density, low levels of large host density cannot sustain the infection (Fig. 5). When the infection persists in the tick population, the densities of infected ticks follow similar trends to those seen for the respective susceptible classes. However, the densities of infected and recovered small hosts now increase and saturate as large host densities increase to high levels and the densities of infected and recovered large hosts increase due to the increased contact with infected ticks. The density of susceptible and infected questing adult ticks decreases as large host density increases to high levels due to the increased availability of large hosts for attachment. Note, the density of infected hosts is small as both host types have a relatively high rate of recovery from infection.

We plot the prevalence of infection in ticks and their hosts for changes in host density (Fig. ). At very low densities of either host type the infection cannot be sustained. For host densities where infection persists, increases in small host density for fixed large host density lead to an increase in prevalence for each individual tick class and host type, which then saturates at high small host density. Initial increases in large host density lead to an increase in total tick prevalence followed by a slight decrease to constant levels at high large host density. At low large host densities the total tick population has a larger proportion of adult ticks, which have a higher prevalence than larvae or nymphs, and so the total tick prevalence is higher than when there is a high large host population and the proportion of adult ticks in the total population is reduced. In general, low densities of one host type and medium densities of the other host correspond to regions of low seroprevalence in hosts. Medium densities of either host type, or low densities of one and high densities of the other, correspond to regions of medium seroprevalence in hosts and high densities in either host type correspond to regions of high host seroprevalence. A key aspect of our model results is that considerable variation in the prevalence of infection in hosts can arise due to changes in host community composition.

**Fig. 6:**
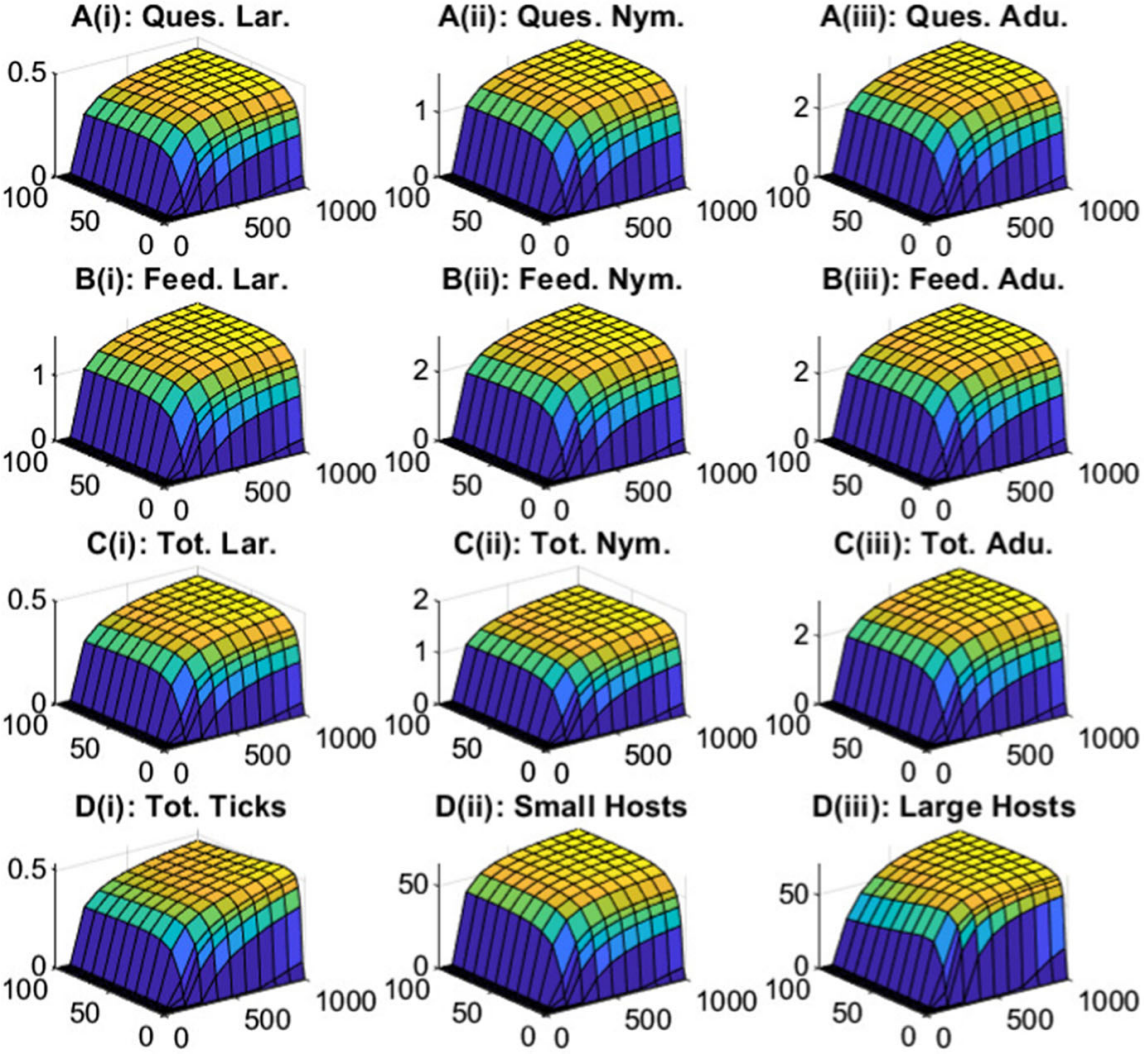
Steady state prevalence and seroprevalence (%) levels for ticks and hosts when varying the large host density (from 0 to 100) and small host density (from 0 to 1000) for the tick-host model with infection represented by Eqs. 3–5. The prevalence levels are shown for **A** questing ticks, **B** feeding ticks and **C** total ticks of a given stage with **D** showing (i) total tick prevalence, (ii) small host seroprevalence and (iii) large host seroprevalence. In plots (A-C) the prevalence levels of the (i) larvae, (ii) nymph and (iii) adult stages of tick are shown. When not varied in the figure parameters are as in Table 1 and 2. Note, the axes scales may differ between subplots

### The Impact of Host Density on Tick and Host Pathogen Epidemiology Under Different Baseline Parasitisation Index Levels

We consider changes in the parasitisation index levels on the epidemiological dynamics and following the methods described in Sect. 2.2 we vary both small host density and large host density for five different sets of parasitisation index levels (Figures S.12 - S.16). The results for different parasitisation index levels are qualitatively similar to the baseline results (see Fig. 6) and, in particular, considerable variation in infection prevalence in both host types is observed for changes in host density. Differences are that the infection prevalence, in both ticks and hosts, increases as the parasitisation index increases, and that for low parasitisation index levels the threshold host density for which the infection can persist is increased. For low $PI_S$, we observe an increase and then decrease in prevalence as large host density is increased (Figure S.12). For further details see Sect.S.3.

### Model Sensitivity to Transmission Parameters

We explore the effect of varying the tick-host transmission coefficient, vertical transmission coefficient and co-feeding transmission coefficient on the tick-host epidemiological dynamics. Here, we vary one transmission coefficient whilst keeping the others fixed at baseline value (Table 2). When varying the tick-host transmission coefficients, we scale all the tick to small host transmission coefficients ($q_L, q_{N1}$) by a factor $q_1$, and all the tick to large host transmission coefficients ($q_{N2}, q_A$) by a factor $q_2$. Increasing any of the transmission coefficients increases the density of infected individuals within the system and reduces the threshold in host density for the infection to persist (Figures S.17 - S.24). At the densities considered in this work the infection cannot persist at low levels of vertical transmission or at low levels of tick to small host transmission (Figures S.17, S.18, S.21 and S.22). However, the infection can persist in the absence of tick to large host transmission or co-feeding transmission (Figures S.19, S.20, S.23 and S.24). For further details see section S.4.

## Discussion

In this paper, we have developed a mathematical model that examines tick and host demographic and epidemiological dynamics when the development of specific tick stages is linked to the specific host types on which they feed. We have shown that a bottleneck can occur that limits tick density increases when the density of one host type increases with fixed levels in the other host. The demographic dynamics have important consequences for the epidemiological dynamics and we show that changes in host community composition (changes in the density of the different host types and the average tick burden of each host type) can lead to a wide range of variation in the prevalence of infection in ticks and hosts.

Previous model studies linking host density to tick density have shown that the relationship between ticks and hosts can have a significant impact on the population dynamics (Cobbold et al. 2015; Lou and Wu 2014; Rosá and Pugliese 2007). Lou et al. (2014) (Lou and Wu 2014) showed that when tick attachment was a frequency dependent function of host density, tick density was constant for changes in host density. When attachment was density-dependent or an increasing saturating function of tick density then adult tick density increased whereas larvae and nymph density initially increased and then decreased as the small mammal density increased. In contrast to our model, the model of Lou et al. (2014) (Lou and Wu 2014) does not have a regulation through attachment that depends on tick density and therefore has no limit to the number of ticks an individual can harbour. Instead, limits on tick density occurred through self-regulation on the birth rate and so as the small host density increases tick births can decrease but attachment rates can increase and saturate. This causes the more rapid progression through the larvae and nymph classes and so the observed decrease in density of these classes is a result of tick self-regulation and not from a potential limit on the maximum number of ticks attached to each host. Cobbold et al. (2015) (Cobbold et al. 2015) argues that each host should contain a maximum number of ticks (Brunner et al. 2013) and that this would implicitly regulate tick density. They considered a framework in which specific tick stages attach to specific hosts but did not examine how changes to total host density could affect tick density. Instead, they considered how changes in the relative proportion of hosts within a specific type would influence tick density. They showed that for increased host biodiversity to lower tick densities (Ostfeld and Keesing 2000), competition among the hosts had to be direct. They suggested that the changes in tick-host encounter rates are a key process that can determine whether increasing biodiversity will regulate tick populations. Rosá and Pugliese (2007) (Rosá and Pugliese 2007) considered different tick stages which feed on different hosts and showed that when the density of ticks is dependent upon total, combined host density (rather than linking the progression of specific tick stages to specific host densities), tick density will continue to increase for an increase in density of one host type and fixed density in the other. A key result of our model study (Sect. 2), where the density of ticks is dependent upon host density and the development stages of ticks are dependent upon a specific host type, is that saturation of tick density can occur due to restrictions caused by a fixed host type when the other host increases in density. Moreover, this saturation can occur at faster or slower rates, and higher or lower densities, depending on the parasitisation index of each host type. This shows that host community composition can be an important driver of tick density and should be considered in tick-host models.

Our results highlighting how tick development linked to specific host density can impact the tick population dynamics will also have important consequences for the epidemiological dynamics of tick-borne infections. In agreement with previous studies (Lou and Wu 2014; Norman et al. 1999; Rosá and Pugliese 2007) we find that at low levels of host density the system cannot support a sufficient density of ticks for infection to be maintained. As host density increases the infection can be supported and initially the level and prevalence of infection increases. For further increases in host density, studies of Norman et al. (1999) and Rosá and Pugliese (2007) observe a dilution effect where the prevalence of infection decreased. In Norman et al. (1999) (Norman et al. 1999) this is due to the increase in non-viraemic host density which does not lead to further infection transmission and so dilutes the pool of infected hosts. For Rosá and Pugliese (2007) (Rosá and Pugliese 2007) dilution arises as the increase in host density is more rapid than the increase in tick density, due to the density dependent function used for tick births. In our model study, increases in host density lead to increases in the prevalence of infection in ticks and hosts with the prevalence saturating at high host densities and so we do not observe a dilution effect at high host density. Our study therefore emphasises how the processes that determine tick regulation can have an important influence on the dynamics of tick-borne infections.

**Fig. 7:**
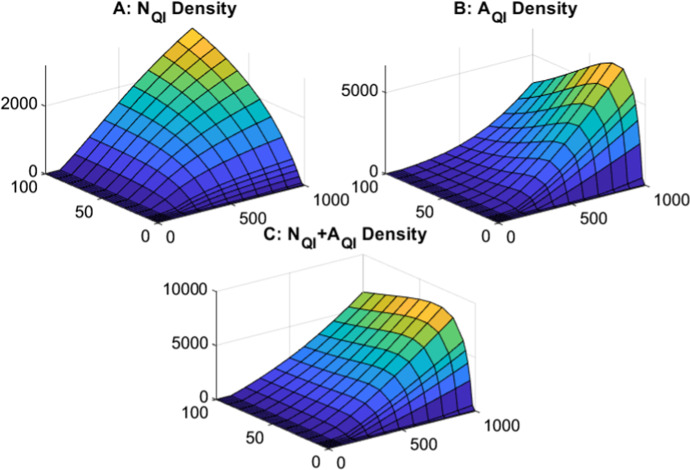
Infected questing tick population densities for varying large host densities (from 0 to 100) and varying small host densities (from 0 to 1000) for the tick-host model with infection represented by Eqs. 3–5. Densities are shown for **A** infected questing nymphs, **B** infected questing adults and **C** infected questing nymphs and adults combined. When not varied in the figure parameters are as in Table 1 and 2

A key aspect of our model study is that host community composition has a significant impact on the prevalence of infection in both the tick and host populations. Our model study highlights how different host densities and different tick burdens on each host type can drive variation in the prevalence and risk of tick-borne disease. This could help to understand and explain the variance in host infection levels seen for different tick-borne diseases, such as Lyme disease in the UK (Cairns et al. 2019), overall tick-borne pathogen prevalence in Europe and China (Grochowska et al. 2020; Zhao et al. 2021), for tick-borne infection in small mammals in Mongolia (Pulscher et al. 2018) and of Crimean-Congo Haemorrhagic Fever virus in ungulates in Spain (Spengler et al. 2019; Cuadrado-Matías et al. 2021). Our study suggests that, at average parasitisation index levels, regions of low disease incidence in either host correspond to regions with low densities of one host type and medium densities of the other. Regions of medium disease incidence correspond to regions with low densities of one host type and high densities of the other, or medium densities in both hosts. Regions of high disease incidence correspond to regions with high densities of both host types. Our model results also indicate that regional variation in the prevalence of infection in hosts could result from regional differences in the average tick burden of host species, with a higher average tick burden leading to higher host infection prevalence. Notwithstanding, regions of high infection prevalence generally correspond to regions of medium or high host density.

There are few theoretical studies which examine the density of infected questing and feeding ticks separately instead of focusing on the density of total infected ticks (but see (Rosá and Pugliese 2007)). By separating each tick stage into a questing and feeding class we can examine the risk of zoonotic spillover, where an increased density of questing infected nymphs or adults would pose a greater risk of pathogen spillover (Eisen et al. 2002). Our model results indicate that increases in small host density lead to increases in the density of questing infected nymphs and adults (Fig.  and see Figures S.25 - S.29 for comparable results with different parasitisation index levels), due to increased rates of progression through the larval and nymphal stage. This is in line with field studies that have indicated a positive correlation between small host density and questing nymph tick densities (Krawczyk et al. 2020), suggesting that high small host densities could lead to the greatest risk of zoonotic spillover to humans or livestock. Initial increases in large host density leads to increases in infected questing nymph and adult ticks (Fig. 7) since this increases overall tick density and therefore tick reproduction. Field studies have also indicated that increases in large host density can lead to an increase in density of infected questing nymphs (Dickinson et al. 2020; Takumi et al. 2019). In our model study further increases in large host density leads to an increase in available hosts for nymph and adult tick attachment and this leads to a reduction in questing nymph and adult ticks suggesting a reduced risk for human and livestock spillover at high large host densities. This process in our model is similar to a ‘mopping effect’ in field situations, where acaricide treated hosts, such as sheep, are allowed to increase in density to provide alternative hosts for ticks, and therefore reduce the risk of transmission to a focal host (Braks et al. 2016). Therefore, our model results indicate that regions with high densities of small hosts, average densities of large hosts and host species with high average tick burden pose the greatest risk of infection spillover to livestock and humans. This highlights the importance of considering host community composition when modelling the epidemiology of host tick systems.

In general, our model framework has been able to explain variation in host prevalence due to changes in specific host density and host tick burden and therefore suggests that host community composition may play a crucial role in explaining the variation in prevalence of tick-borne infection. The model framework can be adapted to represent specific host-tick-infection systems and so can be used to manage tick-borne infections and the spread of zoonotic tick-borne disease to humans and livestock.

## Supplementary Information

Below is the link to the electronic supplementary material.

Supplementary file 1 (pdf 2155 KB)
