# Supplementary material for: The Impact of Host Abundance on the Epidemiology of Tick-Borne Infection
Source: Bull Math Biol. 2023 Mar 9;85(4):30. doi: 10.1007/s11538-023-01133-8 (PMC9998325; doi:10.1007/s11538-023-01133-8)
Supplement: Supplementary file 1 — (pdf 2155 KB) [file 11538_2023_1133_MOESM1_ESM.pdf]

Supplementary Information for The impact of host community composition on the epidemiology of tick-borne infection

S.1 The effect of host density on tick density under different baseline parasitisation index levels

We use the model developed in Equations (1) to explore the effect of different parasitisation index levels (average tick burden per host), for variation in both small and large host density, on tick demography. We consider parasitisation index levels of $5, 10$ and 20 per small host (PI_S) and $5, 40$ and 80 per large host (PI_L), and recalibrate the values of the attachment coefficients $\beta_1, \beta_2, \beta_3, \beta_4$ and s_1, s_2, s_3, s_4, for the different values of PI_S and PI_L. We vary small host density at fixed large host density for five different sets of parasitisation index levels (Figures S.1 - S.5) and vary large host density at fixed small host density for the same five sets of parasitisation index levels (Figures S.6-S.10). The different sets of PI correspond to scenarios with low PI_S and baseline PI_L (Figures S.1 and S.6), baseline PI_S and low PI_L (Figures S.2 and S.7), baseline values of PI_S and PI_L (Figures S.3 and S.8), baseline PI_S and high PI_L (Figures S.4 and S.9), and high PI_S and baseline PI_L (Figures S.5 and S.10).

An increase in the parasitisation index levels leads to an increase in tick density (compare Figures S.1, S.3 and S.5; Figures S.2, S.3 and S.4; Figures S.6, S.8 and S.10; Figures S.7, S.8 and S.9). For increasing small host density, and fixed large host density (Figures S.1 - S.5), a higher ratio of $PI_S : PI_L$ results in a faster saturation in tick densities (compare Figures S.1 to S.2), since the limitation in available large hosts occurs at lower small host density. Similarly, for increasing large host density, and fixed small host density (Figures S.6-S.10), a higher ratio of $PI_L : PI_S$ results in a faster saturation in tick densities (compare Figures S.7 to S.6).

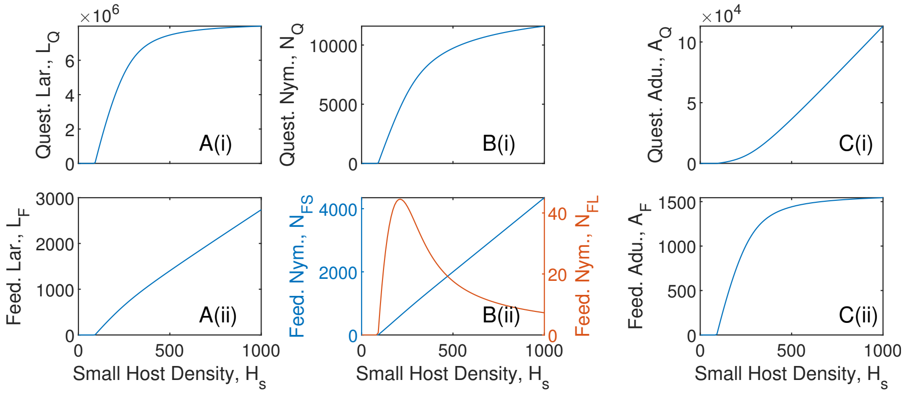

Figure S.1: Same as Figure 2 seen in the main paper, but with $\beta_1 = 0.0000002, \beta_2 = 0.00012, \beta_3 = 0.000094, \beta_4 = 0.0013$ and $s_1 = 0.00000012, s_2 = 0.00013, s_3 = 0.000013, s_4 = 0.00028$. These parameters represent parasitisation index levels of $PI_S = 5$ and $PI_L = 40$.

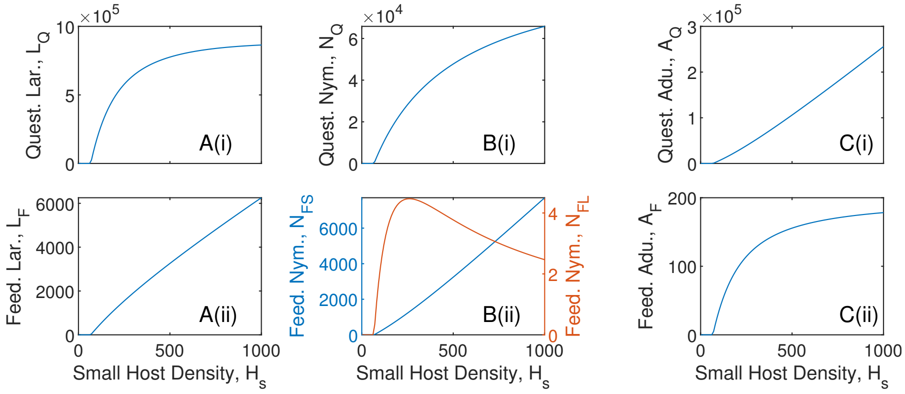

Figure S.2: Same as Figure 2 seen in the main paper, but with $\beta_1 = 0.000004, \beta_2 = 0.000035, \beta_3 = 0.0000018, \beta_4 = 0.000021$ and $s_1 = 0.0000012, s_2 = 0.000019, s_3 = 0.0000019, s_4 = 0.000036$. These parameters represent parasitisation index levels of $PI_S = 10$ and $PI_L = 5$.

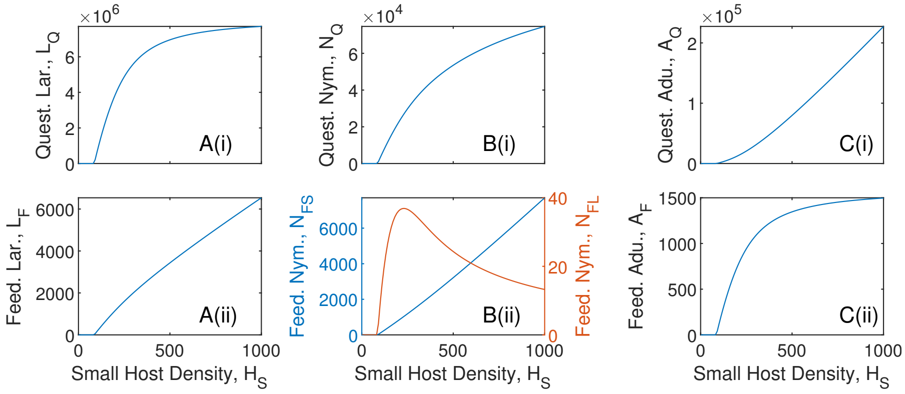

Figure S.3: Same as Figure 2 seen in the main paper, where $PI_S = 10$ and $PI_L = 40$.

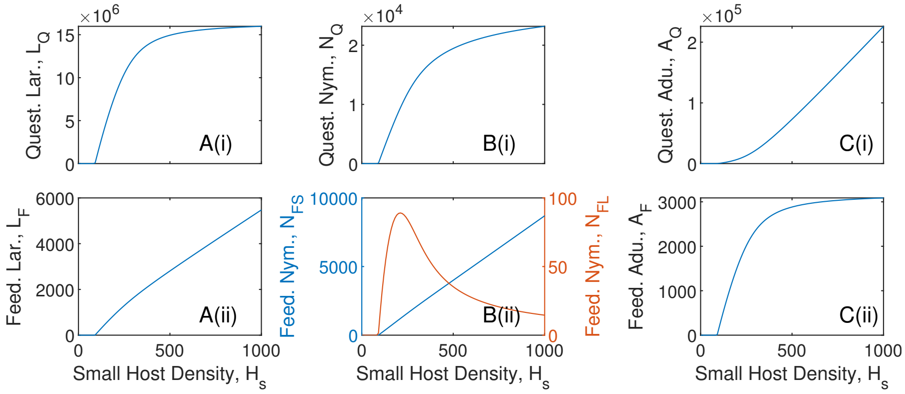

Figure S.4: Same as Figure 2 seen in the main paper, but with $\beta_1 = 0.0000002, \beta_2 = 0.00012, \beta_3 = 0.000094, \beta_4 = 0.0013$ and $s_1 = 0.000000059, s_2 = 0.000064, s_3 = 0.0000064, s_4 = 0.00014$. These parameters represent parasitisation index levels of $PI_S = 10$ and $PI_L = 80$.

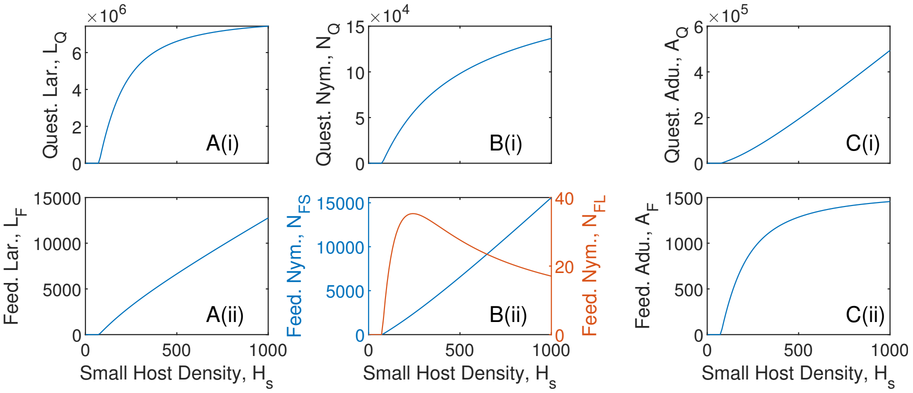

Figure S.5: Same as Figure 2 seen in the main paper, but with $\beta_1 = 0.00000098, \beta_2 = 0.000036, \beta_3 = 0.0000071, \beta_4 = 0.00011$ and $s_1 = 0.00000015, s_2 = 0.0000098, s_3 = 0.00000098, s_4 = 0.000023$. These parameters represent parasitisation index levels of $PI_S = 20$ and $PI_L = 40$.

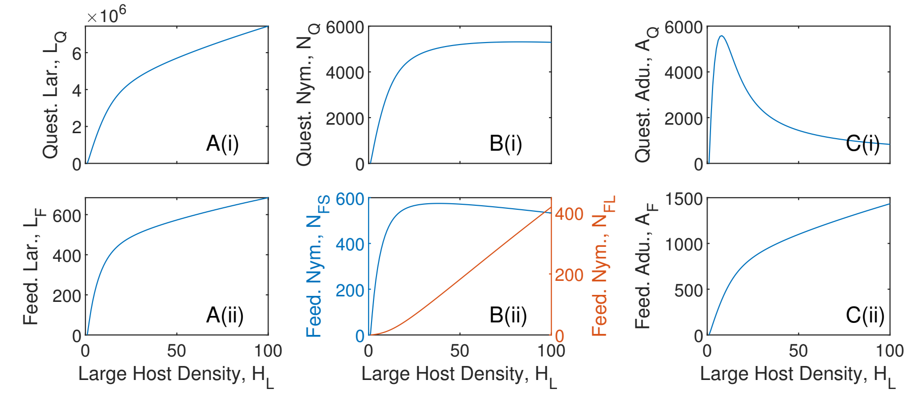

Figure S.6: Same as Figure 3 seen in the main paper, but with $\beta_1 = 0.0000002, \beta_2 = 0.00012, \beta_3 = 0.000094, \beta_4 = 0.0013$ and $s_1 = 0.00000012, s_2 = 0.00013, s_3 = 0.000013, s_4 = 0.00028$. These parameters represent parasitisation index levels of $PI_S = 5$ and $PI_L = 40$.

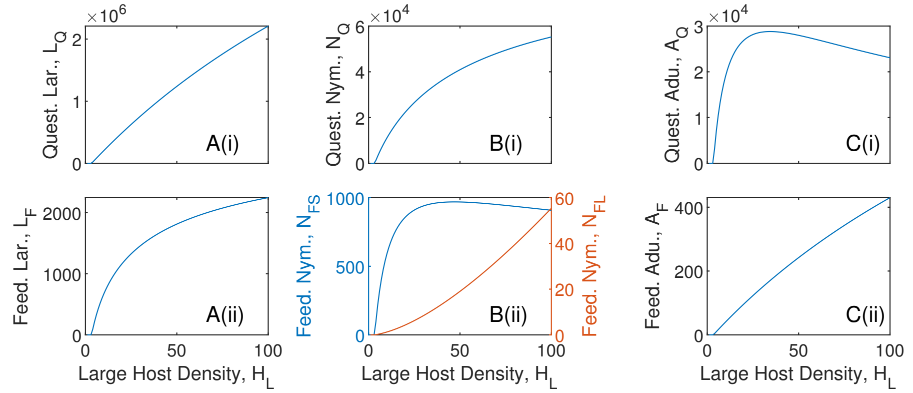

Figure S.7: Same as Figure 3 seen in the main paper, but with $\beta_1 = 0.000004, \beta_2 = 0.000035, \beta_3 = 0.0000018, \beta_4 = 0.000021$ and $s_1 = 0.0000012, s_2 = 0.000019, s_3 = 0.0000019, s_4 = 0.000036$. These parameters represent parasitisation index levels of $PI_S = 10$ and $PI_L = 5$.

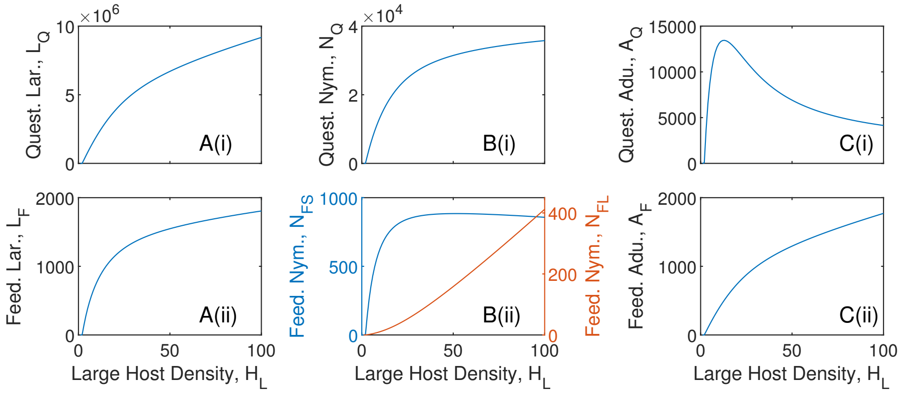

Figure S.8: Same as Figure 3 seen in the main paper, where $PI_S = 10$ and $PI_L = 40$.

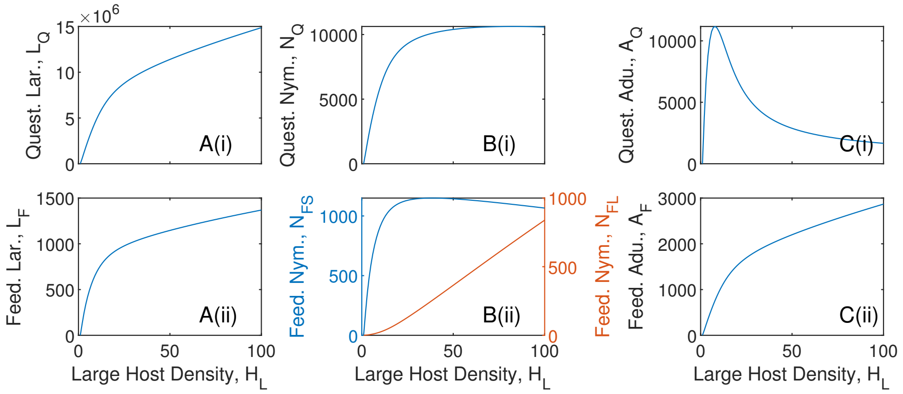

Figure S.9: Same as Figure 3 seen in the main paper, but with $\beta_1 = 0.0000002, \beta_2 = 0.00012, \beta_3 = 0.000094, \beta_4 = 0.0013$ and $s_1 = 0.000000059, s_2 = 0.000064, s_3 = 0.0000064, s_4 = 0.00014$. These parameters represent parasitisation index levels of $PI_S = 10$ and $PI_L = 80$.

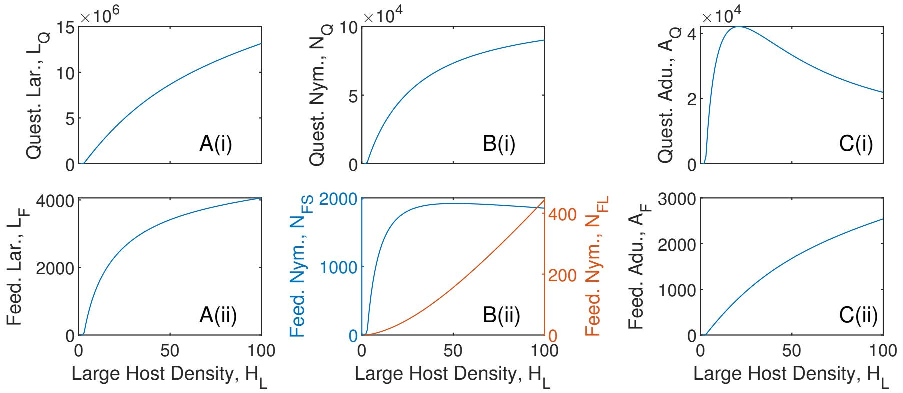

Figure S.10: Same as Figure 3 seen in the main paper, but with $\beta_1 = 0.00000098, \beta_2 = 0.000036, \beta_3 = 0.0000071, \beta_4 = 0.00011$ and $s_1 = 0.00000015, s_2 = 0.0000098, s_3 = 0.00000098, s_4 = 0.000023$. These parameters represent parasitisation index levels of $PI_S = 20$ and $PI_L = 40$.

S.2 Tick-host epidemiological dynamics: model schematic

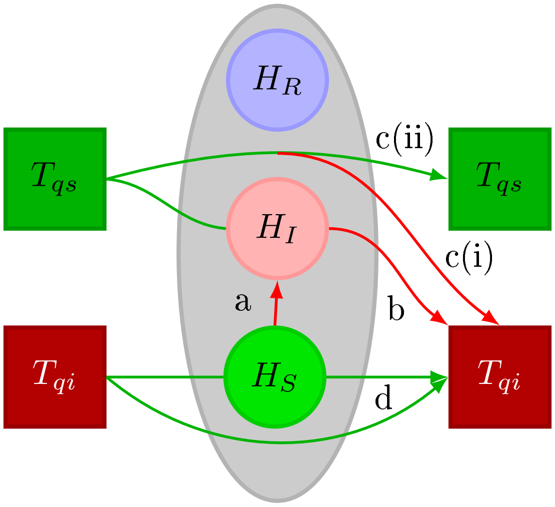

Figure S.11: A schematic diagram detailing the routes of transmission for the tick-host epidemiological model (equations 3-5). In this schematic diagram we consider a representative tick class and host class to highlight the key transmission routes of infection. Square nodes represent the tick classes, and oval/circle nodes represent host classes. The infection status is given by the colour of the node, with green representing a susceptible class, red an infected class and blue a recovered class. The grey ellipse represents the total host population, $H = H_S + H_I + H_R$. Arrows indicate the progression of a tick to the next stage through feeding (green) or infection transmission for ticks or hosts (red). The arrow labelled (a) represents tick-host transmission where a susceptible host becomes infected through contact with an infected tick, (b) represents host-tick transmission where a susceptible tick becomes infected through contact with an infected host, (c) represents susceptible ticks feeding on hosts, highlighting the possibility of infection through co-feeding, c(i), or that ticks may progress to the next stage without contracting infection, c(ii), and (d) which highlights that infected ticks remain infected on progression to the next tick stage. In addition to these transmission routes there is also the possibility of vertical transmission, where births from infected, fed adults can lead to infected offspring.

S.3 The effect of host density on host and tick epidemiology under different baseline parasitisation index levels

We use the model developed in Equations (3-5) to explore the effect of different parasitisation index levels, for variation in both small and large host density, on tick and host epidemiology. As in section S.1, we consider parasitisation indices of $5, 10$ and 20 per small host (PI_S) and $5, 40$ and 80 per large host host (PI_L) and recalibrate the values of the attachment coefficients, $\beta_1, \beta_2, \beta_3, \beta_4$ and s_1, s_2, s_3, s_4, for the different values of PI_S and PI_L. We vary both small and large host density for five different sets of parasitisation index levels (Figures S.12 - S.16). The different sets of PI correspond to scenarios with low PI_S and baseline PI_L (Figure S.12), baseline PI_S and low PI_L (Figure S.13), baseline values of PI_S and PI_L (Figure S.14), baseline PI_S and high PI_L (Figure S.15), and high PI_S and baseline PI_L (Figure S.16).

When the average tick burden on either host is below the baseline value the seroprevalence in hosts is reduced (compare Figures S.12 and S.13 to Figure S.14). Similarly, an increase in the average tick burden on either host above the baseline values leads to an increase in the seroprevalence in

hosts (compare Figures S.15 and S.16 to Figure S.14). The threshold value in host density for which the infection can persist is increased as the parasitisation index decreases. However, the threshold density at which ticks can persist is not sensitive to changes in parasitisation index. For increases in the density of one host type, while the other remains fixed, the density of susceptible ticks that feed on the varying host type increases, peaks and then decreases to a constant level. For average and high parasitisation index values the density of infected ticks follow a similar trend, and so the prevalence of infection in ticks and hosts increases and then saturates. For low parasitisation index values the pathogen cannot establish at low host densities. When the infection can persist, the density of infected ticks still follow a similar trend to the susceptible ticks, but with a 'shift' in density levels (see Figure 5F). This means that at low parasitisation index levels, where there is considerable difference in the threshold between tick persistence and the persistence of infection, the prevalence of infection in ticks and hosts increases, peaks and then decreases to constant levels (see Figure S.12).

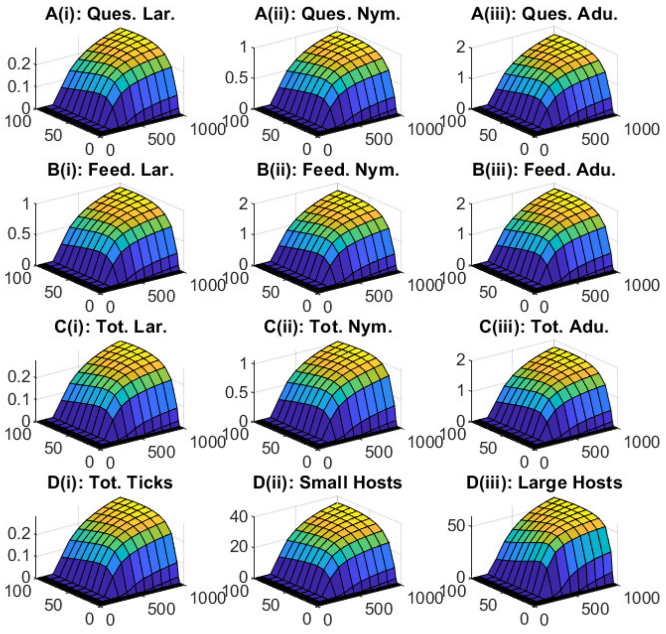

Figure S.12: Same as Figure 6 seen in the main paper, but with $\beta_1 = 0.0000002, \beta_2 = 0.00012, \beta_3 = 0.000094, \beta_4 = 0.0013$ and $s_1 = 0.00000012, s_2 = 0.00013, s_3 = 0.000013, s_4 = 0.00028$. These parameters represent parasitisation index levels of $PI_S = 5$ and $PI_L = 40$.

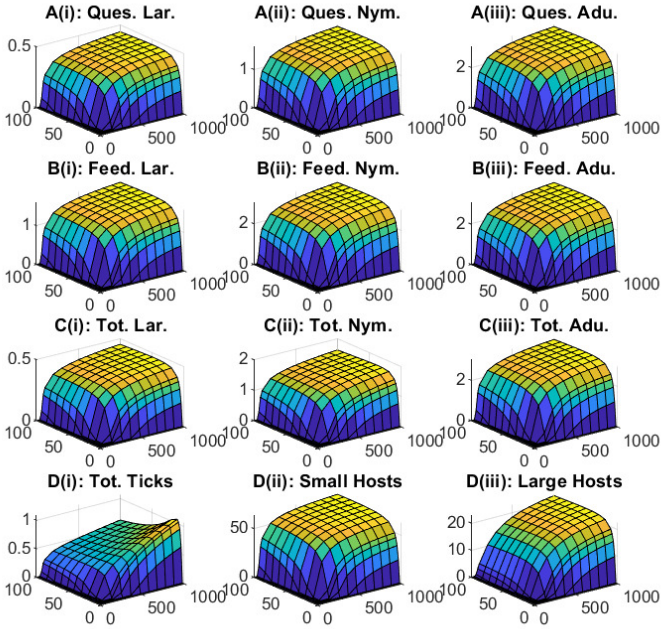

Figure S.13: Same as Figure 6 seen in the main paper, but with $\beta_1 = 0.000004, \beta_2 = 0.000035, \beta_3 = 0.0000018, \beta_4 = 0.000021$ and $s_1 = 0.0000012, s_2 = 0.000019, s_3 = 0.0000019, s_4 = 0.000036$. These parameters represent parasitisation index levels of $PI_S = 10$ and $PI_L = 5$.

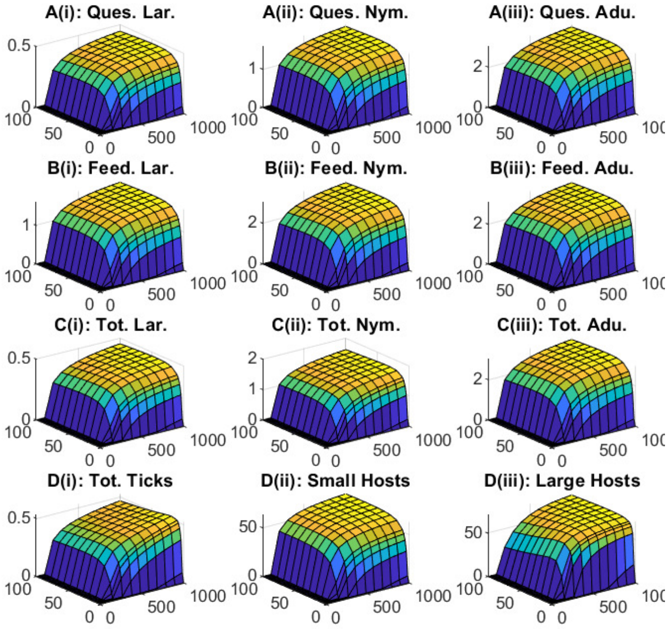

Figure S.14: Same as Figure 6 seen in the main paper, where $PI_S = 10$ and $PI_L = 40$.

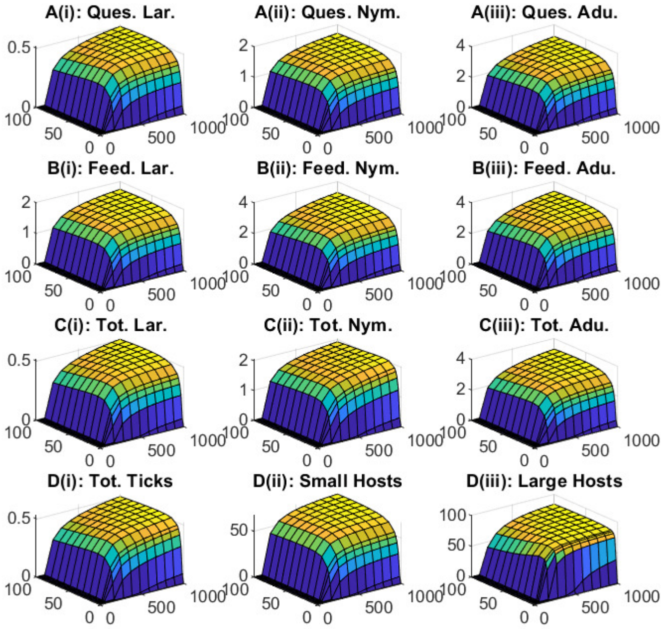

Figure S.15: Same as Figure 6 seen in the main paper, but with $\beta_1 = 0.0000002, \beta_2 = 0.00012, \beta_3 = 0.000094, \beta_4 = 0.0013$ and $s_1 = 0.000000059, s_2 = 0.000064, s_3 = 0.0000064, s_4 = 0.00014$. These parameters represent parasitisation index levels of $PI_S = 10$ and $PI_L = 80$.

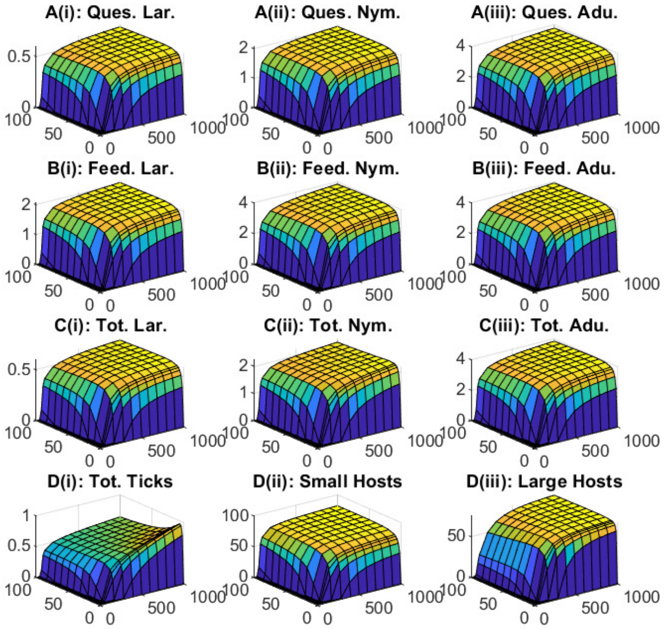

Figure S.16: Same as Figure 6 seen in the main paper, but with $\beta_1 = 0.00000098, \beta_2 = 0.000036, \beta_3 = 0.0000071, \beta_4 = 0.00011$ and $s_1 = 0.00000015, s_2 = 0.0000098, s_3 = 0.00000098, s_4 = 0.000023$. These parameters represent parasitisation index levels of $PI_S = 20$ and $PI_L = 40$.

S.4 Transmission parameter sensitivity

We explore the effect of varying the tick-host transmission coefficient, vertical transmission coefficient and co-feeding transmission coefficient on the tick-host epidemiological dynamics. Here, we vary one transmission coefficient whilst keeping the others fixed at baseline values (see Table 2). When varying the tick-host transmission coefficients, we scale all the tick to small host transmission coefficients by a factor, q_1, and all the tick to large host transmission coefficients by a factor q_2. Each transmission coefficient is varied by the same proportion in relation to its baseline parameter value. We plot model results for four values of each parameter: (i) the absence of that particular transmission route, (ii) half of the baseline parameter value, (iii) the baseline parameter value and (v) 1.5$\times$ the baseline value.

Figures S.17, S.18, S.19 and S.20 show the tick-host epidemiological model results for fixed large host density and varying small host density with varying vertical transmission (Figure S.17), varying tick-host transmission (Figures S.18 and S.19) and varying co-feeding transmission (Figure S.20).

Figures S.21, S.22, S.23 and S.24 show the tick-host epidemiological model results for fixed small host density and varying large host density with varying vertical transmission (Figure S.21), varying tick-host transmission (Figure S.22 and S.23) and varying co-feeding transmission (Figure S.24).

Increasing any of the transmission coefficients increases the density of infected individuals within the system and reduces the threshold in host density for the infection to persist (see Figures S.17-S.24). At the densities considered in this work the infection cannot persist at low levels of vertical transmission or at low levels of tick to small host transmission (Figures S.17, S.18, S.21 and S.22). However, the infection can persist in the absence of tick to large host transmission or co-feeding transmission (Figures S.19, S.20, S.23 and S.24).

When varying the vertical and tick to small host transmission coefficients the simulation results exhibit larger variation in prevalence when compared to the co-feeding or tick to large host transmission coefficients, indicating a stronger model sensitivity for these model parameters (see Figures S.17, S.18, S.21 and S.22). Moreover, varying the tick to large host transmission coefficient has only a minor effect on the epidemiological dynamics of ticks (see Figures S.19 and S.23). For low levels of tick to small host transmission the variation in infected tick density is high, with this increase in tick density decreasing as the transmission coefficient increases further (Figure S.18 and S.22). Increases in vertical transmission or co-feeding transmission result in a consistent increase in infected tick density, therefore showing little variation in sensitivity for these model parameters (Figures S.17, S.20, S.21 and S.24).

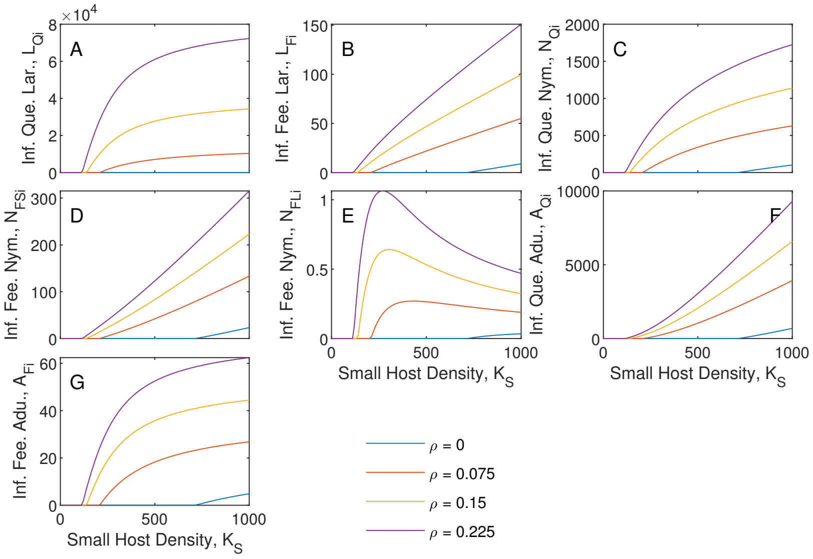

Figure S.17: Infected tick population densities and host seroprevalence levels for a varying small host density and fixed large host density, $H_L = 20$, for the tick-host model framework with infection represented by equations 3-5. Steady state densities were plotted for a varying vertical transmission coefficient with (dotted line) $\rho = 0$, (dot-dashed line) $\rho = 0.085$, (dashed line) $\rho = 0.17$ and (solid line) $\rho = 0.255$ and for (A) questing larvae, (B) feeding larvae, (C) questing nymph, (D) feeding nymphs on small hosts, (E) feeding nymphs on large hosts, (F) questing adults and (G) feeding adults. When not varied in the figure parameter values are as in Table 1 and 2.

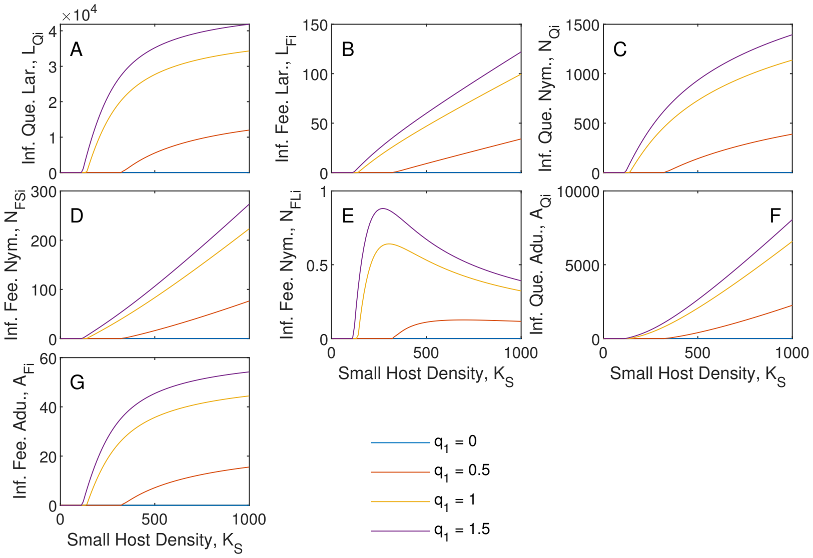

Figure S.18: Infected tick population densities and host seroprevalence levels for a varying small host density and fixed large host density, $H_L = 20$, for the tick-host model framework with infection represented by equations 3-5. Steady state densities were plotted for a varying tick to small host transmission coefficient factor with (dotted line) $q_1 = 0$, (dot-dashed line) $q_1 = 0.5$, (dashed line) $q_1 = 1$ and (solid line) $q_1 = 1.5$ and for (A) questing larvae, (B) feeding larvae, (C) questing nymph, (D) feeding nymphs on small hosts, (E) feeding nymphs on large hosts, (F) questing adults and (G) feeding adults. When not varied in the figure parameter values are as in Table 1 and 2.

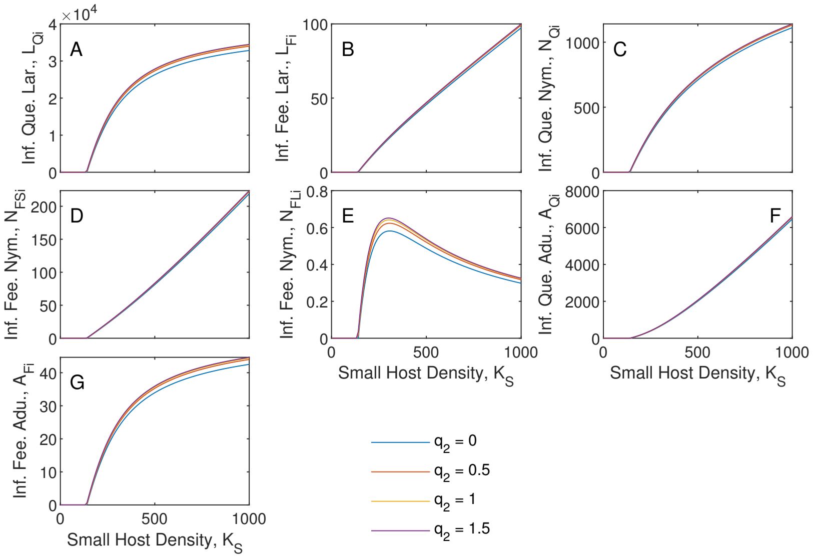

Figure S.19: Infected tick population densities and host seroprevalence levels for a varying small host density and fixed large host density, $H_L = 20$, for the tick-host model framework with infection represented by equations 3-5. Steady state densities were plotted for a varying tick to large host transmission coefficient factor with (dotted line) $q_2 = 0$, (dot-dashed line) $q_2 = 0.75$, (dashed line) $q_2 = 1$ and (solid line) $q_2 = 1.5$ and for (A) questing larvae, (B) feeding larvae, (C) questing nymph, (D) feeding nymphs on small hosts, (E) feeding nymphs on large hosts, (F) questing adults and (G) feeding adults. When not varied in the figure parameter values are as in Table 1 and 2.

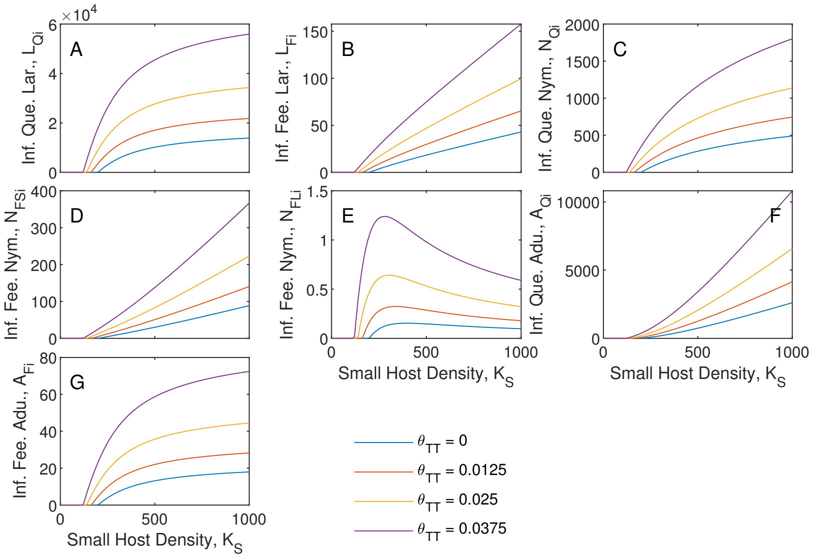

Figure S.20: Infected tick population densities and host seroprevalence levels for a varying small host density and fixed large host density, $H_L = 20$, for the tick-host model framework with infection represented by equations 3-5. Steady state densities were plotted for a varying co-feeding transmission coefficient with (dotted line) $\theta_{TT} = 0$, (dot-dashed line) $\theta_{TT} = 0.0125$, (dashed line) $\theta_{TT} = 0.025$ and (solid line) $\theta_{TT} = 0.0375$ and for (A) questing larvae, (B) feeding larvae, (C) questing nymph, (D) feeding nymphs on small hosts, (E) feeding nymphs on large hosts, (F) questing adults and (G) feeding adults. When not varied in the figure parameter values are as in Table 1 and 2.

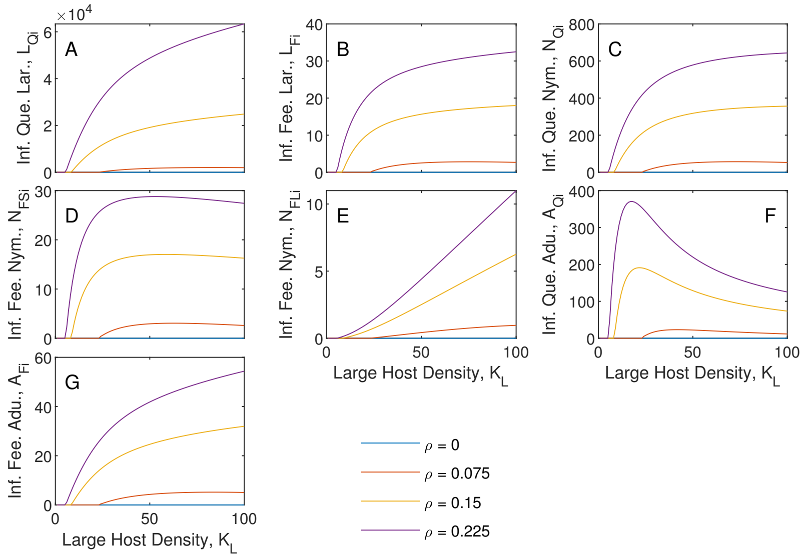

Figure S.21: Infected tick population densities and host seroprevalence levels for a varying large host density and fixed small host density, $H_S = 200$, for the tick-host model framework with infection represented by equations 3-5. Steady state densities were plotted for a varying vertical transmission coefficient with (dotted line) $\rho = 0$, (dot-dashed line) $\rho = 0.085$, (dashed line) $\rho = 0.17$ and (solid line) $\rho = 0.255$ and for (A) questing larvae, (B) feeding larvae, (C) questing nymph, (D) feeding nymphs on small hosts, (E) feeding nymphs on large hosts, (F) questing adults and (G) feeding adults. When not varied in the figure parameter values are as in Table 1 and 2.

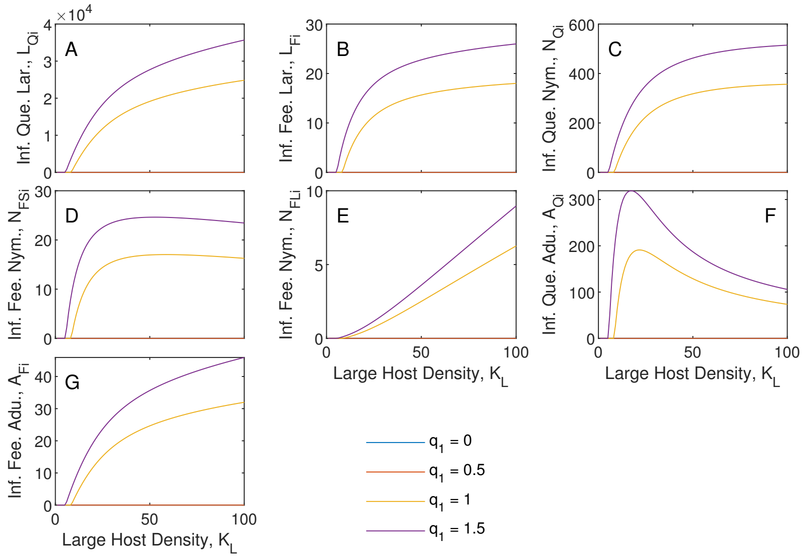

Figure S.22: Infected tick population densities and host seroprevalence levels for a varying large host density and fixed small host density, $H_S = 200$, for the tick-host model framework with infection represented by equations 3-5. Steady state densities were plotted for a varying tick to small host transmission coefficient factor with (dotted line) $q_1 = 0$, (dot-dashed line) $q_1 = 0.5$, (dashed line) $q_1 = 1$ and (solid line) $q_1 = 1.5$ and for (A) questing larvae, (B) feeding larvae, (C) questing nymph, (D) feeding nymphs on small hosts, (E) feeding nymphs on large hosts, (F) questing adults and (G) feeding adults. When not varied in the figure parameter values are as in Table 1 and 2.

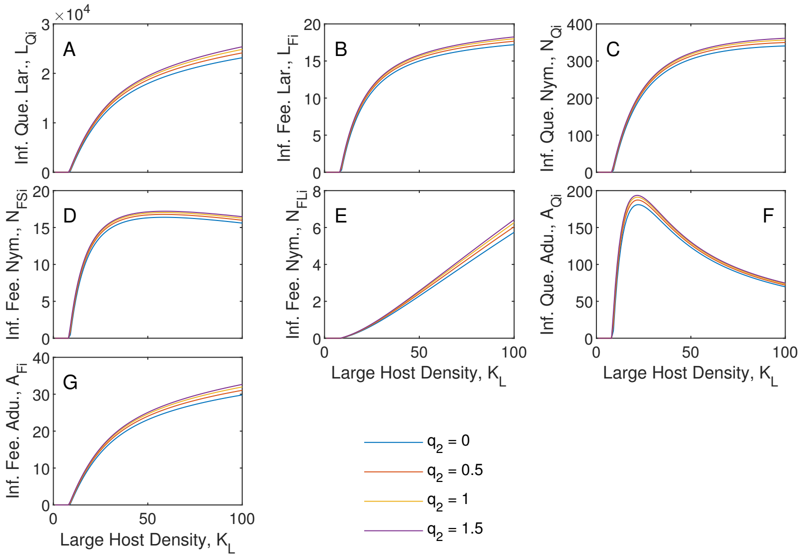

Figure S.23: Infected tick population densities and host seroprevalence levels for a varying large host density and fixed small host density, $H_S = 200$, for the tick-host model framework with infection represented by equations 3-5. Steady state densities were plotted for a varying tick to large host transmission coefficient factor with (dotted line) $q_2 = 0$, (dot-dashed line) $q_2 = 0.5$, (dashed line) $q_2 = 1$ and (solid line) $q_2 = 1.5$ and for (A) questing larvae, (B) feeding larvae, (C) questing nymph, (D) feeding nymphs on small hosts, (E) feeding nymphs on large hosts, (F) questing adults and (G) feeding adults. When not varied in the figure parameter values are as in Table 1 and 2.

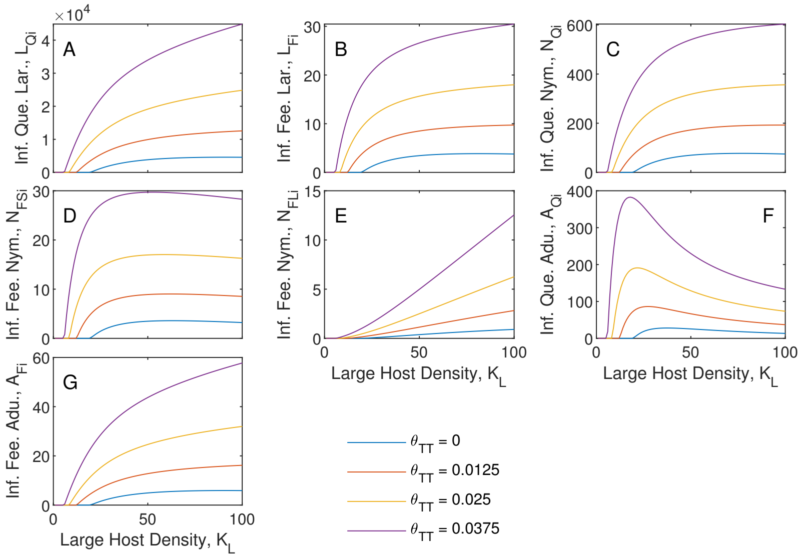

Figure S.24: Infected tick population densities and host seroprevalence levels for a varying large host density and fixed small host density, $H_S = 200$, for the tick-host model framework with infection represented by equations 3-5. Steady state densities were plotted for a varying co-feeding transmission coefficient with (dotted line) $\theta_{TT} = 0$, (dot-dashed line) $\theta_{TT} = 0.0125$, (dashed line) $\theta_{TT} = 0.025$ and (solid line) $\theta_{TT} = 0.0375$ and for (A) questing larvae, (B) feeding larvae, (C) questing nymph, (D) feeding nymphs on small hosts, (E) feeding nymphs on large hosts, (F) questing adults and (G) feeding adults. When not varied in the figure parameter values are as in Table 1 and 2.

S.5 Risk of pathogen spillover

By separating each tick stage into a questing and feeding class we can examine the risk of zoonotic spillover, where an increased density of questing infected nymphs or adults would pose a greater risk of pathogen spillover [18]. Here we produce model simulation results that show the densities of infected questing nymph ticks, infected questing adult ticks and total infected nymph and adult ticks, for different host compositions. We vary both small and large host density for five different sets of parasitisation index levels (Figures S.25 - S.29). The different sets of PI correspond to scenarios with low PI_S and baseline PI_L (Figure S.25), baseline PI_S and low PI_L (Figure S.26), baseline values of PI_S and PI_L (Figure S.27), baseline PI_S and high PI_L (Figure S.28), and high PI_S and baseline PI_L (Figure S.29).

When the total average tick burden across the system is increased, the number of infected questing nymph, adult and total ticks is increased, therefore increasing the risk of pathogen spillover (compare Figures S.25 and S.28, where the PI ratio between small and large hosts is equivalent). Here, the system can support more ticks, consequently increasing the number of questing ticks. As the tick burden on small hosts is increased, at fixed tick burden on large hosts, the range of host densities for which the risk of pathogen spillover is greatest is increased (compare Figures S.25, S.27 and S.29). Here, the regulation arising from small hosts is not as strong, reducing the rate at which the infected questing nymph and adult tick densities decrease and saturate. However, as the tick burden on large hosts is increased, for fixed tick burden on small hosts, the range of host densities for which the risk of pathogen spillover is greatest is decreased (compare Figures S.26, S.27 and S.28). Here, the regulation arising from the fixed small host density is more pronounced, which increases the rate at which the infected questing nymph and adult tick densities decrease and saturate.

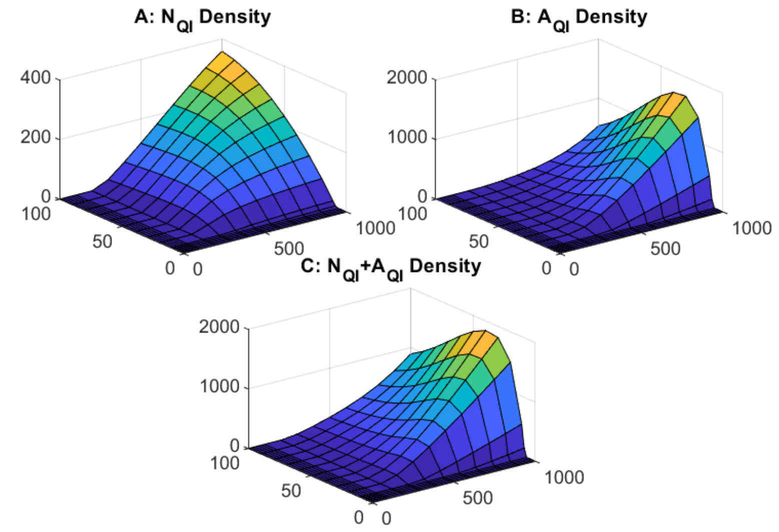

Figure S.25: Infected questing tick population densities for varying large host densities (from 0 to 100) and varying small host densities (from 0 to 1000), for the tick-host model framework with infection (equations 3-5), with $PI_S = 5$ and $PI_L = 40$. Densities are shown for (A) infected questing nymphs, (B) infected questing adults and (C) infected questing nymphs and adults combined. Attachment coefficients take values $\beta_1 = 0.0000002, \beta_2 = 0.00012, \beta_3 = 0.000094, \beta_4 = 0.0013$ and $s_1 = 0.00000012, s_2 = 0.00013, s_3 = 0.000013, s_4 = 0.00028$. When not varied in the figure all other parameters are as in Table 1 and 2.

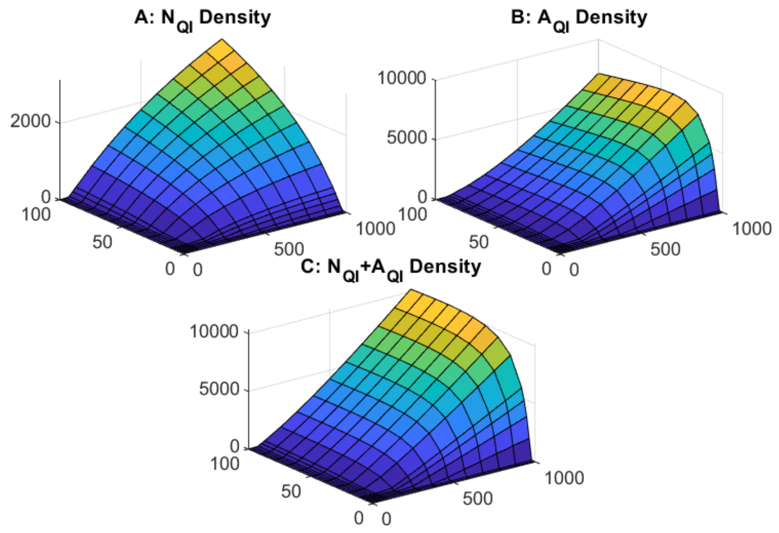

Figure S.26: Infected questing tick population densities for varying large host densities (from 0 to 100) and varying small host densities (from 0 to 1000), for the tick-host model framework with infection (equations 3-5), with $PI_S = 10$ and $PI_L = 5$. Densities are shown for (A) infected questing nymphs, (B) infected questing adults and (C) infected questing nymphs and adults combined. Attachment coefficients take values $\beta_1 = 0.000004, \beta_2 = 0.000035, \beta_3 = 0.0000018, \beta_4 = 0.000021$ and $s_1 = 0.0000012, s_2 = 0.000019, s_3 = 0.0000019, s_4 = 0.000036$. When not varied in the figure all other parameters are as in Table 1 and 2.

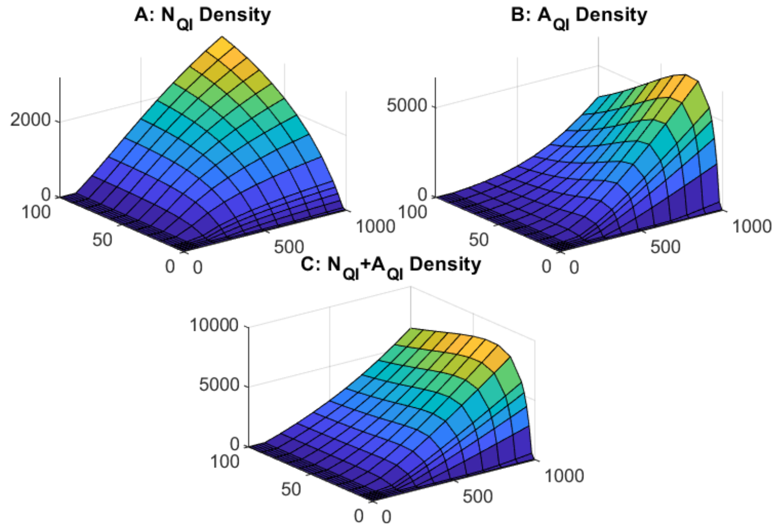

Figure S.27: Same as Figure 7 seen in the main paper, where $PI_S = 10$ and $PI_L = 40$.

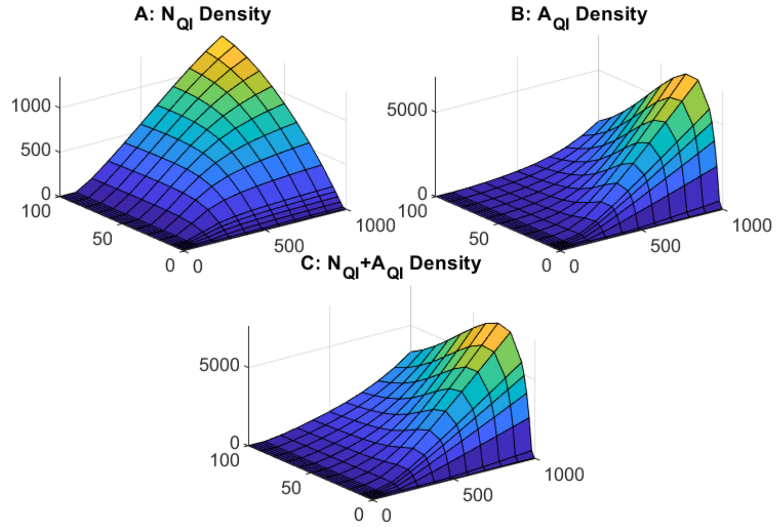

Figure S.28: Infected questing tick population densities for varying large host densities (from 0 to 100) and varying small host densities (from 0 to 1000), for the tick-host model framework with infection (equations 3-5), with $PI_S = 10$ and $PI_L = 80$. Densities are shown for (A) infected questing nymphs, (B) infected questing adults and (C) infected questing nymphs and adults combined. Attachment coefficients take values $\beta_1 = 0.0000002, \beta_2 = 0.00012, \beta_3 = 0.000094, \beta_4 = 0.0013$ and $s_1 = 0.000000059, s_2 = 0.000064, s_3 = 0.0000064, s_4 = 0.00014$. When not varied in the figure all other parameters are as in Table 1 and 2.

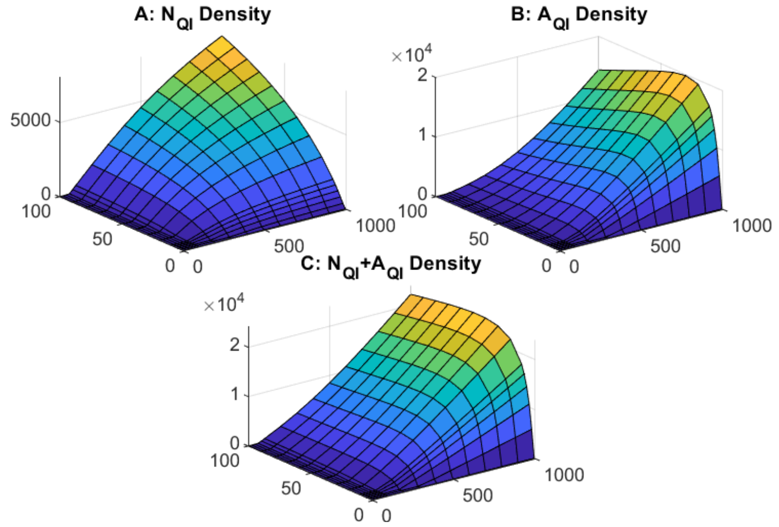

Figure S.29: Infected questing tick population densities for varying large host densities (from 0 to 100) and varying small host densities (from 0 to 1000), for the tick-host model framework with infection (equations 3-5), with $PI_S = 20$ and $PI_L = 40$. Densities are shown for (A) infected questing nymphs, (B) infected questing adults and (C) infected questing nymphs and adults combined. Attachment coefficients take values $\beta_1 = 0.00000098, \beta_2 = 0.000036, \beta_3 = 0.0000071, \beta_4 = 0.00011$ and $s_1 = 0.00000015, s_2 = 0.0000098, s_3 = 0.00000098, s_4 = 0.000023$. When not varied in the figure all other parameters are as in Table 1 and 2.
